# Assembly and regulation of the mammalian mRNA processing body

**DOI:** 10.1371/journal.pone.0282496

**Published:** 2023-03-06

**Authors:** Donald B. Bloch, Claire O. Sinow, Andrew J. Sauer, Benjamin H. P. Corman

**Affiliations:** 1 Division of Rheumatology, Allergy and Immunology, Department of Medicine, Massachusetts General Hospital and Harvard Medical School, Boston, Massachusetts, United States of America; 2 Anesthesia Center for Critical Care Research, Department of Anesthesia, Critical Care, and Pain Medicine, Massachusetts General Hospital and Harvard Medical School, Boston, Massachusetts, United States of America; Wake Forest School of Medicine: Wake Forest University School of Medicine, UNITED STATES

## Abstract

Messenger RNA processing bodies (P-bodies) are cytoplasmic membrane-free organelles that contain proteins involved in mRNA silencing, storage and decay. The mechanism by which P-body components interact and the factors that regulate the stability of these structures are incompletely understood. In this study, we used a fluorescence-based, two-hybrid assay to investigate interactions between P-body components that occur inside the cell. LSm14a, PATL1, XRN1, and NBDY were found to interact with the N-terminal, WD40-domain-containing portion of EDC4. The N-terminus of full-length PATL1 was required to mediate the interaction between EDC4 and DDX6. The C-terminal, alpha helix-domain- containing portion of EDC4 was sufficient to mediate interaction with DCP1a and CCHCR1. In the absence of endogenous P-bodies, caused by depletion of LSm14a or DDX6, expression of the portion of EDC4 that lacked the N-terminus retained the ability to form cytoplasmic dots that were indistinguishable from P-bodies at the level of UV light microscopy. Despite the absence of endogenous P-bodies, this portion of EDC4 was able to recruit DCP1a, CCHCR1 and EDC3 to cytoplasmic dots. The results of this study permit the development of a new model of P-body formation and suggest that the N-terminus of EDC4 regulates the stability of these structures.

## Introduction

Messenger RNA processing bodies (P-bodies) are highly conserved, membrane-free cytoplasmic organelles that regulate post-transcriptional gene expression at the level of mRNA translation and stability (reviewed in [1–3]). Because P-bodies contain proteins involved in removing the 5’ cap from mRNA (DCP2, DCP1A, EDC3, EDC4) and causing 5’ to 3’ degradation of mRNA (XRN1), P-bodies were initially thought to be sites of mRNA catabolism. However, P-bodies also contain proteins that repress translation of mRNA (PATL1, DDX6, LSm14a), suggesting that P-bodies may serve as sites of mRNA silencing and storage. Recent studies showed that P-bodies contain mRNAs that are present in low concentration in cells and encode regulatory, instead of “housekeeping”, proteins [4]. In addition, P-bodies may dissociate in response to external or internal stimuli and release stored mRNAs that are translated, resulting in proteins that produce a coordinated cellular response [4, 5].

In addition to mRNA degradation, silencing and storage, P-body components have other important roles in the cell: MARF1 is required for normal oogenesis, has endonuclease activity and prevents activation of endogenous retroviruses [6–8]; CCHCR1 may have a role in the differentiation of keratinocytes and is involved in the pathogenesis of papillomavirus infection [9–11]; and DDX6 and LSm14a contribute to innate host defense by “sensing” the presence of RNA viruses and regulating the interferon response [12–15]. Despite the various important functions of P-body components, the mechanism by which these proteins interact within P-bodies and the factors that regulate the integrity of these structures are incompletely understood.

Inhibitors of mRNA synthesis cause dissociation of P-bodies, possibly by blocking delivery of mRNA to these structures. In addition, a 69-amino acid microprotein, designated “non-annotated P-body dissociating polypeptide” (or “NBDY”), localizes to P-bodies and, when overexpressed and phosphorylated, results in the disappearance of these structures [16, 17]. Depletion of specific P-body components, including EDC4 [18], LSm14a [19, 20], PATL1 [21, 22] and DDX6 [23, 24] also disrupts P-bodies. Whether each of these proteins is required to physically link P-body components, or whether the function of the proteins is required for P-body integrity, has not yet been determined.

In a previous study, we developed a mammalian two-hybrid assay to investigate interactions between P-body components [25]. The assay is based on the observation that when P-body component EDC4 is fused to an exogenous nuclear localization sequence (NLS) and expressed in mammalian cells, the protein localizes to discrete nuclear, rather than the usual cytoplasmic, dots. We used NLS-EDC4 as “bait” to determine whether other P-body components (“prey”) also localize with NLS-EDC4 in nuclear dots. EDC4 was found to interact with DCP2, DCP1a, MARF1 as well as with itself [8, 25]. EDC3 did not co-localize with NLS-EDC4 in nuclear dots unless DCP1a was also over-expressed, suggesting that endogenous DCP1a was unable to bridge the interaction between NLS-EDC4 and EDC3. The low concentration of DCP1a in the cell (relative to the levels of transiently expressed NLS-EDC4 and EDC3) and/or the presence of a nuclear export signal (NES) in DCP1a may prevent the endogenous protein from facilitating localization of EDC3 to the nucleus. The observation that NLS-EDC4 can only direct EDC3 to nuclear dots when DCP1a is also overexpressed suggests that the two-hybrid assay is able to distinguish between proteins that directly interact and proteins that require one or more additional proteins to mediate interaction.

In earlier studies, we were unable to use the mammalian two-hybrid assay to demonstrate interaction between EDC4 and LSm14a or EDC4 and DDX6 [25]. A potential limitation of the two-hybrid assay is that a strong, endogenous NES in the “prey” protein might dominate over the exogenous NLS fused to EDC4 and thereby prevent co-localization in nuclear dots. In the present study, we overcame the limitation of a strong NES in the prey protein by one of three approaches: 1) removing the endogenous NES; 2) inhibiting nuclear export; or 3) fusing a NLS to the prey protein. We used this modified two-hybrid assay to further investigate interactions between proteins in the P-body, including EDC4, LSm14a, DDX6, PATL1, XRN1, NBDY, CCHCR1, EDC3 and DCP1a. In addition, we confirmed many of the two-hybrid assay results by reconstituting P-bodies in cells that lack these structures. The results of this study permit the development of a new model of protein interactions within mRNA P-bodies and suggest a mechanism by which the integrity of P-bodies is regulated.

## Methods

### Plasmids and antisera

S1 Table in S1 File includes a list of plasmids, antisera and other reagents that were used in this study. S1 Fig depicts the P-body proteins, and fragments of these proteins, that were studied in the two-hybrid assay and in experiments to reconstitute P-body-like structures.

### Cell culture, transfection, immunohistochemistry and co-immunoprecipitation

HEp-2 (catalogue #CCL-23) and HEK293 (CRL-1573) cells were obtained from American Type Cell Collection (Manassas, VA) and maintained in DMEM supplemented with 10% fetal calf serum, L-glutamine (2 mM), penicillin (200 U/ml), and streptomycin (200 ug/ml). Cells were transfected using the Effectene transfection system (Qiagen, Valencia, CA) as directed by the manufacturer. HEp-2 cells were used for immunohistochemistry; twenty-four hours after transfection, cells were fixed with 2% paraformaldehyde in PBS and permeabilized with 0.2% triton X-100 in PBS. Cells were stained with primary and secondary antisera as indicated.

To investigate the interaction between NLS-EDC4 and PATL1, cells were transfected with both plasmids and subsequently treated for 2 hours with leptomycin B (LMB; 10nM) to inhibit nuclear export.

To deplete endogenous P-bodies from HEp-2 cells, cells were transfected with siRNA directed against LSm14a, DDX6, or with scrambled siRNA using Dharmafect (Dharmacon, Lafayette, CO) as directed by the manufacturer. Twenty-four hours after treatment with siRNA, cells were transfected with plasmids encoding P-body components. Cells were subsequently fixed, permeabilized and stained with primary and secondary antisera.

To permit semi-quantitative analysis of the interaction between P-body components in the two-hybrid assay, the number of transfected cells expressing two (or more) P-body components in nuclear dots was compared to the total number of cells expressing two (or more) of the transfected proteins. Three separate transfections were performed and at least 30 cells expressing two (or more) proteins were scored in each transfection. Results are presented as mean +/- SEM.

For co-immunoprecipitation assays, HEK293 cells were transfected with plasmids encoding GFP-EDC4 and monomeric cherry (mCh) fused to XRN1(1232–1706), GFP-EDC4(630–1437 and mCh-XRN1(1232–1706) or with mCh-XRN1(1232–1706) alone. Twenty-four hours after transfection, cells were incubated with lysis buffer containing Tris-HCl pH7.4 (25 mM), NaCl (150 mM), EDTA (1mM), NP-40 (1%) and glycerol (5%). The GFP-containing fusion proteins were immunoprecipitated using GFP-Trap magnetic agarose, as directed by the manufacturer (Proteintech, Rosemont, Illinois). Proteins were eluted from the magnetic agarose by boiling in 2X sample buffer.

## Results

### EDC4 and the two-hybrid assay

Previous studies identified three domains in EDC4 [18, 26, 27]. The N terminus of EDC4 (between amino acids 1–630) contains a WD40 motif (amino acids 131–538), which is predicted to form a seven blade “propeller” structure. The N-terminus also contains a serine rich region between amino acids 611–629 (17 of 19 amino acids are serine), which may serve as a “flexible hinge” between the N and C terminal portions of the protein [27]. The central portion of EDC4 (amino acids 630–935) contains a functional bipartite nuclear localization sequence between amino acids 910–927 [26], suggesting that a small fraction of endogenous, cellular EDC4 may localize to the nucleus. The C terminus of EDC4 (amino acids 935–1437) contains an alpha helix-rich region that mediates interaction with DCP2 and with EDC4 itself [25, 27]. There are two splice variants of EDC4: The longer isoform of EDC4 (Genbank #MW689236), which was used in this study, contains an in-frame insertion of 36 amino acids, beginning at amino acid 1001 of the shorter protein (Genbank #NM_014329).

When EDC4 is forced to enter the nucleus, because of fusion to an exogenous NLS, the protein localizes to nuclear dots [25]. In contrast to NLS-EDC4, fusion of an NLS to other P-body components, including LSm14a, DCP1a or DDX6, causes these proteins to localize diffusely throughout the nucleus (S2 Fig). To investigate the protein motifs required for interactions between EDC4 and cellular structures, as well as between EDC4 and other P-body components, plasmids encoding three portions of EDC4 fused to the NLS of SV40 T antigen (PKKKRK) were prepared (S1 Fig): Full-length EDC4 (amino acids 1–1437); EDC4 lacking the N-terminal WD40 domain and serine-rich region (EDC4 (630–1437)); and EDC4 containing the C-terminal, alpha helix-rich region (amino acids 935–1437).

#### NLS-EDC4 localizes to Cajal bodies

**Fig 1:**
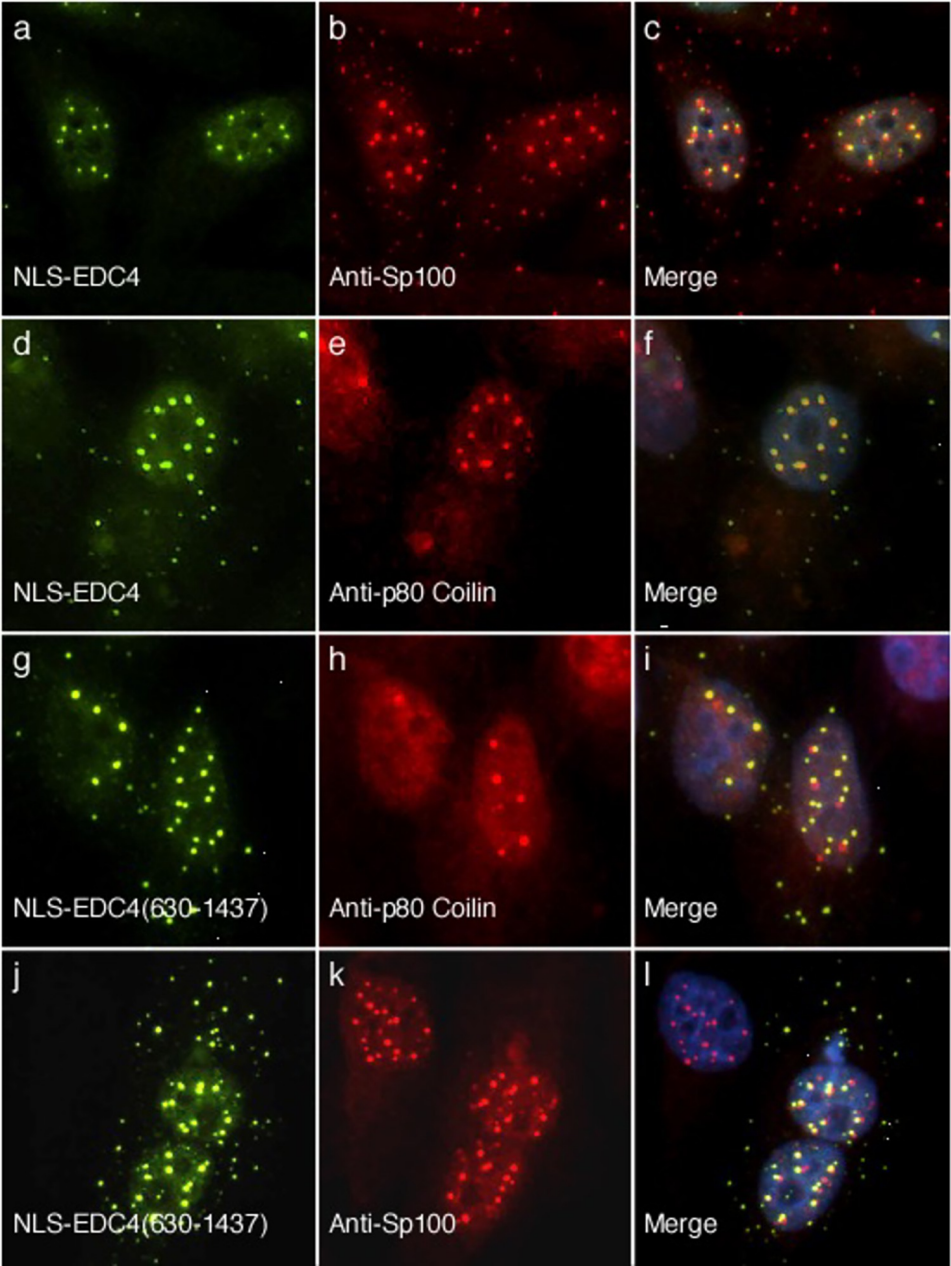
NLS-EDC4 localizes to Cajal bodies, and adjacent to PML-nuclear bodies. A plasmid encoding green fluorescent protein (GFP) fused to the nuclear localization sequence (NLS) of SV40 T antigen and full length EDC4 was transfected into HEp-2 cells. GFP-NLS-EDC4 (panel a) localized adjacent to PML-nuclear bodies (panels b, c). The GFP-NLS-EDC4 fusion protein was detected using mouse monoclonal anti-GFP antibodies and PML nuclear bodies were identified using rabbit anti-Sp100 antiserum. Transfection of GFP-NLS-EDC4 and subsequent staining with mouse anti-GFP antibodies and rabbit anti-p80 coilin antibodies revealed that NLS-EDC4 (panel d) localized to Cajal bodies (panels e, f). Transfection of HEp-2 cells with a plasmid encoding GFP-NLS-EDC4(630–1437) (panel g) and subsequent staining with mouse anti-GFP antibodies and human serum containing anti-p80 coilin antibodies (panels h, i), revealed that in the absence of the N-terminus, EDC4 was no longer able to localize to Cajal bodies. GFP-NLS-EDC4(630–1437) (panel j) retained the ability to localize adjacent to PML nuclear bodies (panels k, l). Merge of panels a and b, d and e, g and h, and j and k is shown in panels c, f, i, and l respectively. DAPI staining (blue) indicates the location of nuclei in c, f, i and l.

To identify the nuclear dots that contain EDC4 when the protein is forced to enter the nucleus, HEp-2 cells were transfected with a plasmid encoding NLS-EDC4 and cells were stained with anti-EDC4 antibodies and rabbit antiserum directed against the Sp100 protein in promyelocytic leukemia (PML) nuclear bodies. NLS-EDC4 localized adjacent to PML-containing nuclear bodies (Fig 1A–1C), as previously reported [28]. Because Cajal bodies are also dot-like structures in the nucleus, we investigated the relationship between NLS-EDC4 and Cajal bodies. NLS-EDC4-transfected cells were stained with human anti-EDC4 antibodies and rabbit antiserum directed against a Cajal body marker protein (p80 coilin). NLS-EDC4 co-localized with p80 coilin in Cajal bodies (Fig 1D–1F). Similar results were obtained when cells were transfected with a plasmid encoding green fluorescent protein (GFP) fused to NLS-EDC4 and cells were subsequently stained with antibodies directed against GFP and human serum containing anti-p80 coilin antibodies (S3A–S3C Fig). Of note, the nuclear distribution of NLS-EDC4 is similar to that of P-body component PATL1, when the nuclear export signal (NES) of PATL1 is inhibited or mutated ([29] and see below).

To determine whether the N-terminus of EDC4, which contains the WD40 domain and serine-rich domain, is required for localization to Cajal bodies, HEp-2 cells were transfected with a plasmid encoding GFP-NLS-EDC4(630–1437) and cells were stained with mouse anti-GFP antibodies and human serum containing anti-p80 coilin antibodies. Although GFP-NLS-EDC(630–1437) localized to nuclear dots, the protein rarely, if ever, localized with p80 coilin in Cajal bodies (Fig 1G–1I). The portion of EDC4 that lacks the N terminal 629 amino acids retained the ability to localize adjacent to PML nuclear bodies (Fig 1J–1L). Taken together, the results show that full-length EDC4, when forced to enter the nucleus, localizes to Cajal bodies and adjacent to PML-containing nuclear bodies. The N-terminus of EDC4 is required for localization to Cajal bodies, but the remainder of the protein is sufficient to mediate localization adjacent to PML nuclear bodies.

### Interaction between EDC4 and PATL1

**Fig 2:**
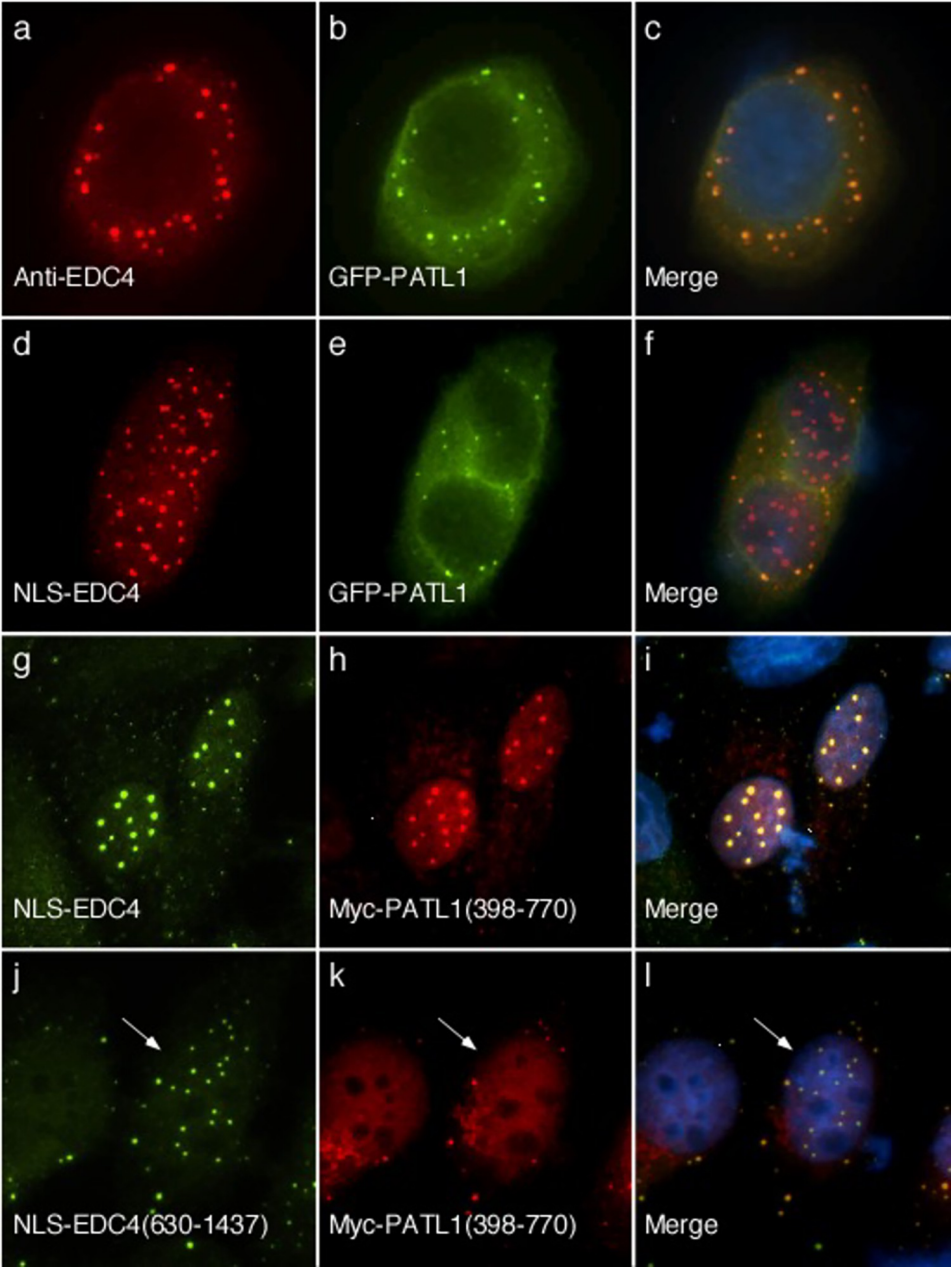
Interaction between PATL1 and EDC4 requires the presence of the C-terminus of PATL1 and the N-terminus of EDC4. After transfection of a plasmid encoding GFP-PATL1 into HEp-2 cells, endogenous EDC4 (panel a) and GFP-PATL1 (panels b, c) localized to cytoplasmic dots. Co-expression of NLS-EDC4 and GFP-PATL1 in HEp-2 cells resulted in localization of NLS-EDC4 (panel d) in nuclear dots, while GFP-PATL1 remained in cytoplasmic dots (panels e, f). Expression of a plasmid encoding NLS-EDC4 (panel g) together with a plasmid encoding Myc fused to PATL1 amino acids 398–770 (panels h, i) resulted in the two proteins co-localizing in nuclear dots in 97 +/-3% of cells that expressed both proteins. When cells were transfected with GFP-NLS-EDC4(630–1437) (panel j) and Myc-PATL1(398–770) (panels k, l), GFP-NLS-EDC4(630–1437) localized to nuclear dots while Myc-PATL1(398–770) remained in cytoplasmic dots in all cells that expressed both proteins. Note that the anti-Myc antiserum produces faint diffuse nuclear staining. EDC4 in panels a, d, and g was detected using human serum containing anti-EDC4 antibodies. GFP-PATL1 in panels b and e, and GFP-NLS-EDC4(630–1437) in panel j were detected using mouse monoclonal anti-GFP antibodies. Myc-PATL1 in panels h and k was detected using rabbit anti-Myc antiserum. Merge of panels a and b, d and e, g and h, and j and k is shown in c, f, i and l respectively. DAPI staining (blue) indicates location of nuclei in c, f, i and I. White arrows in j-l indicate a representative cell that contains NLS-EDC4(630–1437) in nuclear dots, while Myc-PATL1(398–770) localized to cytoplasmic dots.

PATL1 is a P-body component that has important roles in mRNA transport, translation and decay [29]. Transfection of a plasmid encoding GFP fused to PATL1 into HEp-2 cells and subsequent staining with mouse anti-GFP and human anti-EDC4 antibodies confirmed that PATL1 co-localizes with endogenous EDC4 (Fig 2A–2C). Co-expression of plasmids encoding NLS-EDC4 and GFP-PATL1 resulted in localization of EDC4 to nuclear dots, while GFP-PATL1 remained in cytoplasmic dots (Fig 2D–2F). However, previous studies showed that the N-terminus of PATL1 contains a strong NES and that the C-terminus (amino acids 398–770) is sufficient for localization to P-bodies [30]. To further investigate possible interaction between PATL1 and EDC4, plasmids encoding Myc-PATL1(398–770) (which lacks the endogenous NES) and NLS-EDC4 were co-transfected into HEp-2 cells and cells were stained with rabbit anti-Myc antiserum and human serum containing anti-EDC4 antibodies. The C-terminus of PATL1 co-localized with full-length NLS-EDC4 in nuclear dots (Fig 2G–2I). To determine whether the N-terminus of EDC4 is required for interaction with PATL1, cells were transfected with Myc-PATL1(398–770) and GFP-NLS-EDC4(630–1437). GFP-NLS-EDC4(630–1437) localized to nuclear dots while Myc-PATL1(398–770) remained in cytoplasmic dots, suggesting that the N-terminus of EDC4 is required for interaction with PATL1 (Fig 2J–2L).

**Fig 3:**
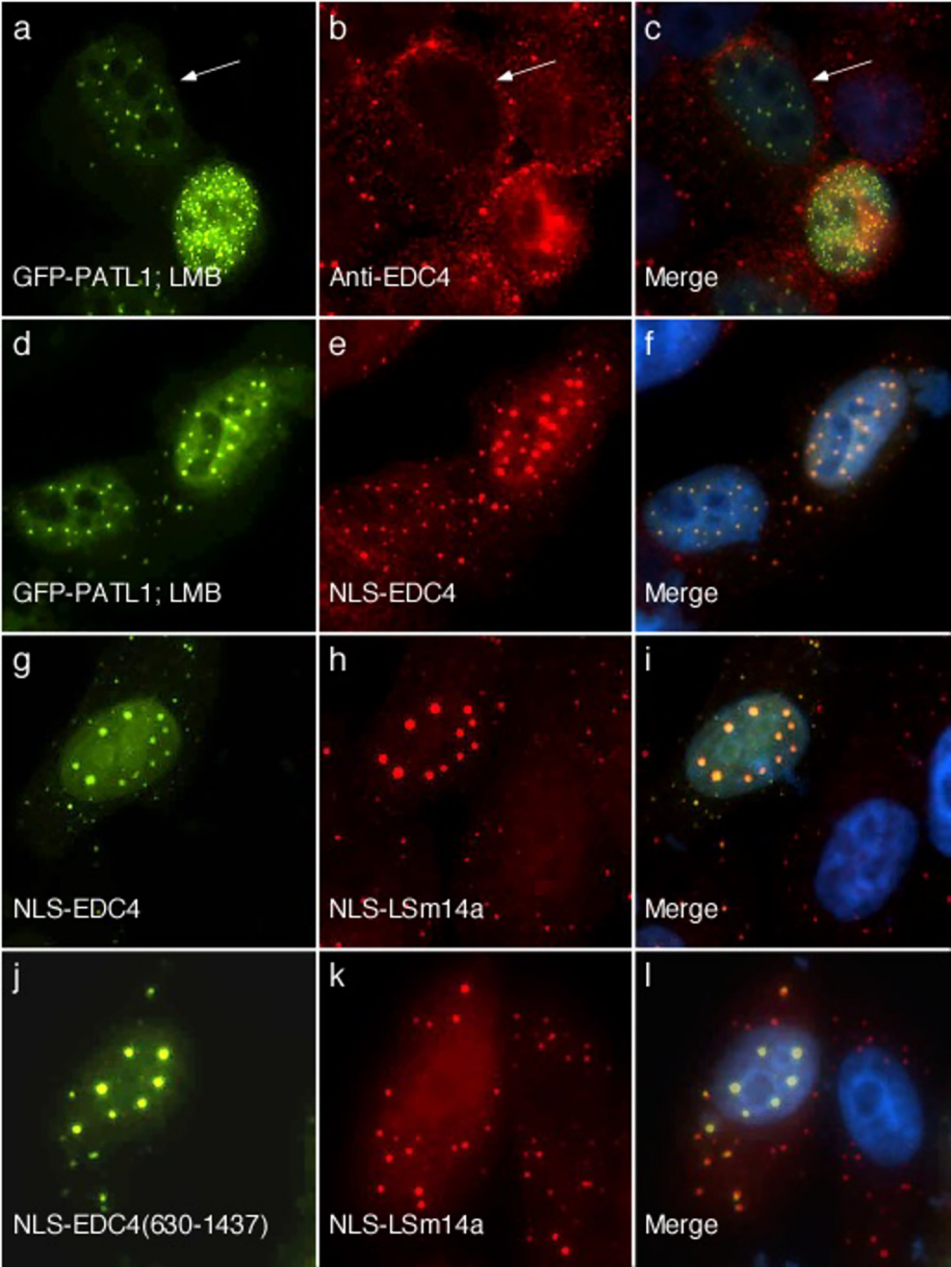
Full-length PATL1 interacts with EDC4. Transfection of HEp-2 cells with GFP-PATL1 followed by treatment with leptomycin B (LMB) resulted in localization of PATL1 in one of three nuclear patterns: homogeneous (not shown), finely speckled and nuclear dot (panel a). Endogenous EDC4 remained in cytoplasmic dots, despite treatment with LMB (panels b, c). White arrows in a-c indicate a representative cell with GFP-PATL1 in nuclear dots, while endogenous EDC4 remained in P-bodies. Co-expression of GFP-PATL1 (panel d) and NLS-EDC4 (panels e, f) and subsequent treatment with LMB resulted in the two proteins localizing to nuclear dots in all cells that expressed both proteins. **NLS-EDC4 recruits NLS-LSm14a to nuclear dots and the N-terminus of EDC4 is required for the observed interaction.** Co-expression of NLS-EDC4 (panel g) and NLS-LSm14a (panel h) resulted in localization of the two proteins to nuclear dots (panel i) in 86 +/-4% of cells with both proteins in the nucleus. In cells transfected with GFP-NLS-EDC4(630–1437) and NLS-LSm14a, GFP-NLS-EDC4(630–1437) was detected in nuclear dots (panel j), while NLS-LSm14a (panel k, l) was distributed diffusely throughout the nucleus. NLS-LSm14a did not co-localize with GFP-NLS-EDC4(630–1437) in nuclear dots in any of the cells that contained both proteins in the nucleus. Mouse monoclonal anti-GFP antibody was used to detect GFP-PATL1 in a and d, and GFP-EDC4(630–1437) in panel j. Human serum was used to detect EDC4 in b, e, and g. Rabbit anti-LSm14a antiserum was used to detect NLS-LSm14a in h and k. Note that the rabbit antiserum detected endogenous LSm14a in cytoplasmic dots (panels h, k). Merge of panels a and b, d and e, g and h, and j and k is shown in c, f, i and l respectively. DAPI staining (blue) indicates location of nuclei in c, f, i, and l.

To investigate whether full-length PATL1, like the C-terminal portion of the protein, is able to interact with EDC4, we took advantage of the observation that full-length PATL1 shuttles between the nucleus and the cytoplasm. Inhibition of nuclear export, using leptomycin B (LMB), increases the amount of PATL1 in the nucleus [30]. When HEp-2 cells were transfected with GFP-PATL1 alone and treated for two hours with LMB, staining for PATL1 revealed three distinct nuclear staining patterns: diffuse (55% of cells), finely speckled (30% of cells) and nuclear dots (15% of cells) (the last two patterns are shown in Fig 3A–3C). Co-transfection of HEp-2 cells with NLS-EDC4 and GFP-PATL1, followed by treatment with LMB for two hours, resulted in co-localization of the proteins in nuclear dots (Fig 3D–3F). In the presence of NLS-EDC4, nuclear PATL1 was only seen in nuclear dots and was not detected in either the diffuse or finely speckled nuclear patterns. The results confirm that full-length PATL1 interacts with EDC4 and suggest that, at least under the conditions tested, NLS-EDC4 appears to determine the nuclear location of PATL1.

### Interaction between EDC4 and LSm14a

LSm14a is a P-body component that is a member of the Sm family of RNA binding proteins [19]. In a previous study using the mammalian two-hybrid assay, we were unable to demonstrate a direct interaction between EDC4 and LSm14a [25]. To consider the possibility that LSm14a contains a NES that is stronger than the interaction between NLS-EDC4 and LSm14a, we prepared a plasmid encoding the SV40 T antigen NLS fused to LSm14a (NLS-LSm14a). When expressed alone in HEp-2 cells, NLS-LSm14a localized diffusely throughout the nucleus (S2G and S2H Fig). When co-expressed with NLS-EDC4, NLS-LSm14a co-localized with NLS-EDC4 in nuclear dots (Fig 3G–3I), suggesting that the two proteins directly interact. To determine whether the N-terminus of EDC4 is required for the protein to interact with LSm14a, plasmids encoding GFP-NLS-EDC4(630–1437) and NLS-LSm14a were transfected into HEp-2 cells. GFP-NLS-EDC4(630–1437) localized to nuclear dots, while NLS-LSm14a was detected diffusely throughout the nucleus and was not concentrated in nuclear dots (Fig 3J–3L). The results show that LSm14a interacts with EDC4 and that the N terminus of EDC4 is required for interaction between the two proteins.

### Interaction between EDC4 and XRN1

**Fig 4:**
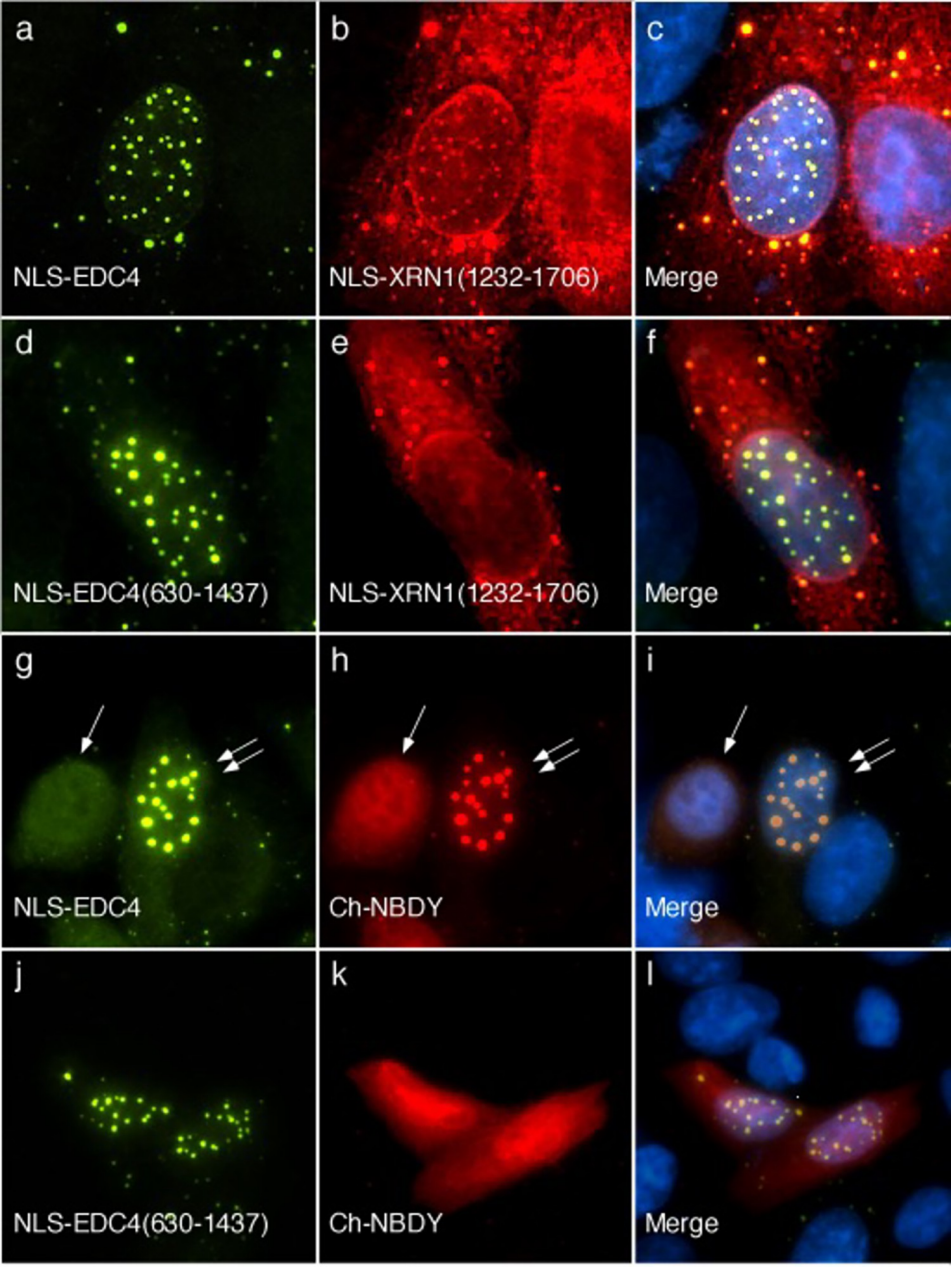
EDC4 interacts with XRN1. Transfection of HEp-2 cells with GFP-NLS-EDC4 (panel a) and monomeric cherry (mCh) fused to SV40 T antigen NLS and XRN1 amino acids 1232–1706 (mCh-NLS-XRN1(1232–1706) (panel b) resulted in localization of both proteins in nuclear dots (panel c) in 94+/-2% of cells expressing both proteins. Co-expression of GFP-NLS-EDC4(630–1437) and mCh-NLS-XRN1(1232–1706) resulted in localization of GFP-NLS-EDC4(630–1437) to nuclear dots (panel d) while mCh-NLS-XRN1(1232–1706) remained in cytoplasmic dots (panels e, f) in all cells that expressed both proteins. **EDC4 interacts with NBDY.** Co-expression of NLS-EDC4 (panel g) and mCh-NBDY (panels h, i) resulted in both proteins localizing diffusely throughout the nucleus in 59+/-2% of cells expressing both proteins. In the remaining cells that expressed both proteins, mCh-NBDY co-localized with NLS-EDC4 in nuclear dots. A single white arrow in g-i indicates a cell expressing both NLS-EDC4 and mCh-NBDY with both proteins in a homogeneous nuclear staining pattern. Double white arrows in g-i indicate a cell with both proteins in nuclear dots. Localization of GFP-NLS-EDC4(630–1437) to nuclear dots (panel j) was not affected by expression of mCh-NBDY (panels k, l). Human serum was used to detect NLS-EDC4 in panels a and g, and mouse monoclonal anti-GFP detected GFP-EDC4(630–1437) in d and j. Rabbit anti-mCh antiserum was used to detect mCh-NLS-XRN1(1232–1706) in b and e and mCh-NBDY in h and k. Merge of panels a and b, d and e, g and h, and j and k is shown in c, f, I and l respectively. DAPI staining (blue) indicates the location of nuclei in c, f, i and l.

XRN1 is a cytoplasmic exoribonuclease that mediates the last step in the 5’ to 3’ mRNA decay pathway. Previous investigators showed that the C-terminus of XRN1 is required for localization to P-bodies [31]. To investigate potential interaction between EDC4 and XRN1 inside cells, a plasmid encoding monomeric cherry (mCh) fluorescent protein fused to XRN1 amino acids 1232–1706 was prepared and expressed in HEp-2 cells. Co-expression of NLS-EDC4 and mCh-XRN1(1232–1706) resulted in localization of NLS-EDC4 to nuclear dots, while mCh-XRN1(1232–1706) localized to cytoplasmic dots (S3D–S3F Fig). To consider the possibility that this portion of XRN1 contains a strong NES, a second plasmid, encoding the same portion of XRN1 fused to the SV40 T antigen NLS (mCh-NLS-XRN1(1232–1706)), was expressed in HEp-2 cells. Even when fused to the exogenous NLS, this portion of XRN1 remained in the cytoplasm (S2I and S2J Fig). However, when mCh-NLS-XRN1(1232–1706) was co-expressed with NLS-EDC4, both proteins localized to nuclear dots (Fig 4A–4C), suggesting that the carboxyl portion of XRN1 does, in fact, interact with EDC4 and that the combination of NLS-EDC4 and the NLS on XRN1(1232–1706) is sufficient to overcome the presumed NES in the carboxyl portion of XRN1.

To determine whether the N-terminal, WD40-containing domain in EDC4 is required for interaction with XRN1, HEp-2 cells were transfected with plasmids encoding mCh-NLS-XRN1(1232–1706) and GFP-NLS-EDC4(630–1437). GFP-NLS-EDC4(630–1437) localized to nuclear dots while mCh-NLS-XRN1(1232–1706) remained in the cytoplasm (Fig 4D–4F). Taken together, the results confirm that EDC4 interacts with XRN1 and show that that the N-terminus of EDC4 is required for interaction with XRN1.

We used a co-immunoprecipitation assay to confirm that the N-terminus of EDC4 is required for interaction with XRN1(1232–1706). HEK293 cells were transfected with mCh-XRN1(1232–1706) alone or GFP-EDC4(630–1437) and mCh-XRN1(1232–1706) or GFP-EDC4 and mCh-XRN1(1232–1806). GFP-Trap magnetic agarose beads were used to immunoprecipitate the GFP fusion proteins. GFP-EDC4, but neither the magnetic beads alone nor GFP-EDC4(630–1437), was able to co-precipitate mCh-XRN1(1232–1706) (S4 Fig).

### Interaction between EDC4 and NBDY

NBDY is a 69-amino acid microprotein that interacts with EDC4 and regulates P-body formation. Transfection of NBDY into mammalian cells decreases the number of cells with P-bodies; however, in some cells that over-express NBDY, P-bodies are not disrupted and NBDY co-localizes with these structures [32]. Na and colleagues used immunoprecipitation and pull-down assays to show that NBDY interacts with the N-terminus of EDC4 [16]. To confirm that NBDY interacts with EDC4 inside cells, NLS-EDC4 and mCh-NBDY were co-expressed in HEp-2 cells. In the majority of cells, expression of mCh-NBDY resulted in loss of NLS-EDC4-containing nuclear dots. In the remaining cells, mCh-NBDY co-localized with NLS-EDC4 in nuclear dots (Fig 4G–4I).

To investigate whether the N-terminus of EDC4 is required for interaction with NBDY, cells were transfected with mCh-NBDY and GFP-NLS-EDC4(630–1437). In cells expressing both proteins, NBDY did not localize to nuclear dots and NBDY had no effect on the nuclear dot distribution of GFP-NLS-EDC4(630–1437) (Fig 4J–4l). The results confirm that the N-terminus of EDC4 is required for interaction with NBDY inside the cell.

### Interaction between EDC4 and DCP1A

**Fig 5:**
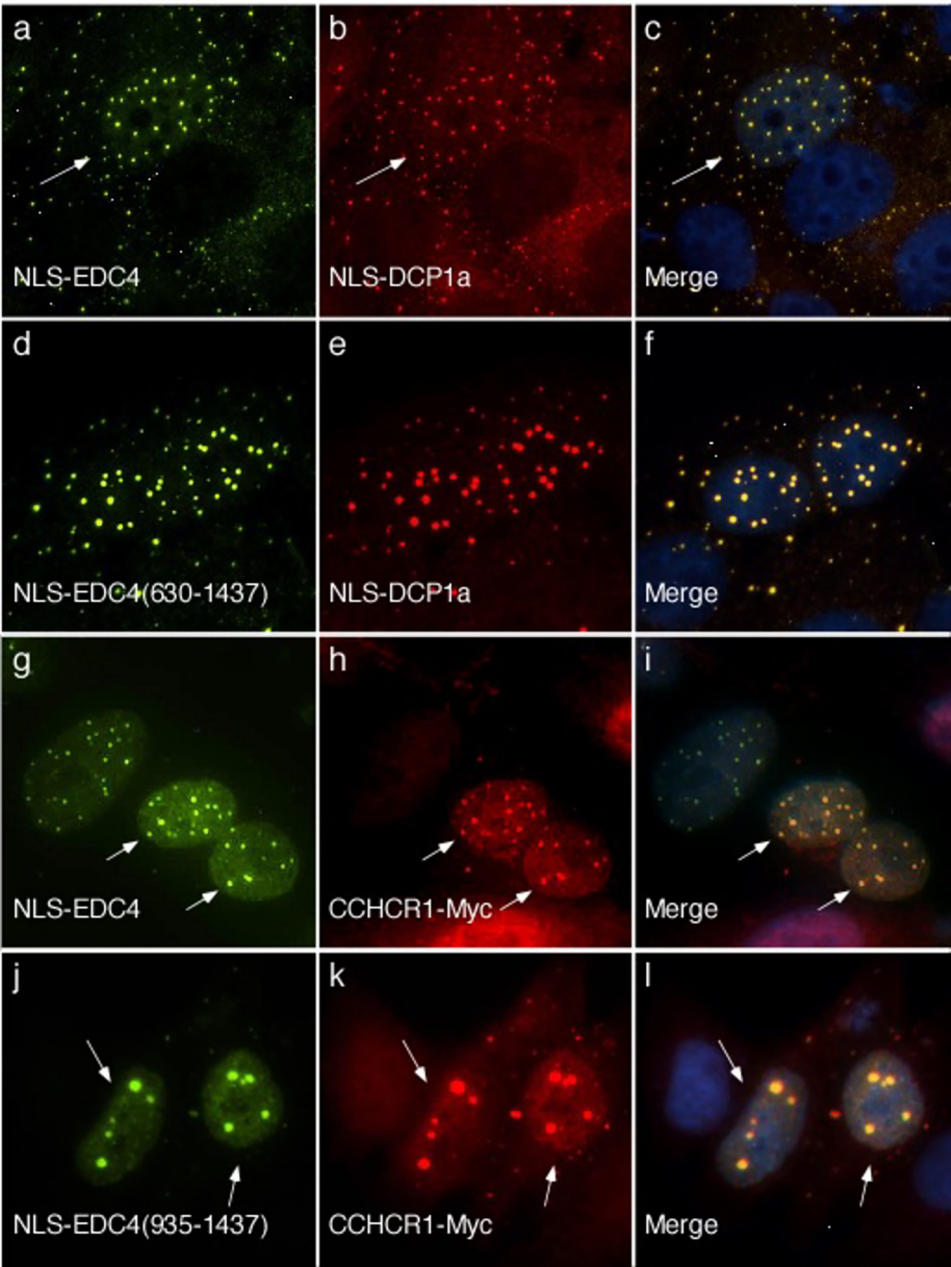
The N-terminus of EDC4 is not required for interaction with DCP1a. Co-expression of NLS-EDC4 (panel a) and NLS-DCP1a (panel b) resulted in co-localization of the proteins to nuclear dots (panel c) in all cells that had both proteins in the nucleus. White arrows in a-c indicate a representative cell containing both NLS-EDC4 and NLS-DCP1a in nuclear dots. GFP-NLS-EDC4(630–1437) (panel d) was also able to recruit NLS-DCP1a (panels e, f) to nuclear dots in all cells that expressed both proteins. **The C-terminus of EDC4 is sufficient to mediate interaction with CCHCR1.** Co-expression of NLS-EDC4 (panel g) and CCHCR1-Myc (panel h) resulted in co-localization of the proteins in nuclear dots (panel i) in all cells that expressed both proteins. The C-terminus of EDC4 (GFP-NLS-EDC4(935–1437)) (panel j) was sufficient to recruit CCHCR1-Myc (panels k, l) to nuclear dots. CCHCR1-Myc co-localized with GFP-NLS-EDC4(935–1437) in 96+/-4% of cells expressing both proteins. White arrows indicate the location of representative cells that expressed NLS-EDC4 and CCHCR1-Myc (g-i) and NLS-EDC4(935–1437) and CCHCR1-Myc (j-l). Human serum was used to detect NLS-EDC4 in a and g. Rabbit antiserum was used to detect NLS-DCP1a in b and e. Rabbit anti-Myc antiserum was used to detect CCHCR1-Myc in h and k. Mouse monoclonal anti-GFP antibody detected GFP-NLS-EDC4(630–1437) and GFP-NLS-EDC4(935–1437) in d and j respectively. Merge of panels a and b, d and e, g and h, and j and k is shown in c, f, i and l respectively. DAPI staining (blue) indicates the location of nuclei in c, f, i and l.

DCP1a enhances the decapping activity of DCP2 and in mammalian cells this interaction requires the presence of EDC4 [33]. To further investigate the interaction between EDC4 and DCP1a, a plasmid encoding the SV40 T antigen NLS fused to DCP1a was prepared. Transfection and expression of NLS-DCP1a in HEp-2 cells resulted in localization of NLS-DCP1a diffusely throughout the nucleus (S2E and S2F Fig). Co-expression of NLS-EDC4 and NLS-DCP1a in HEp-2 cells resulted in co-localization of the two proteins in nuclear dots (Fig 5A–5C). To determine whether the N-terminus of EDC4 is required for interaction with DCP1a, HEp-2 cells were transfected with GFP-NLS-EDC4(630–1437) and NLS-DCP1a. As with NLS-EDC4, GFP-NLS-EDC4(630–1437) was able to direct NLS-DCP1a to nuclear dots (Fig 5D–5F), showing that the N-terminal portion of EDC4 is not required for interaction with DCP1. In a previous study, we observed that co-expression of the C-terminus of EDC4 (amino acids 935–1437) with DCP1a also resulted in the localization of both proteins to nuclear dots. In addition, we previously showed that the C-terminus of EDC4 was sufficient to pull-down full-length DCP1a [25].

### Interaction between EDC4 and CCHCR1

The gene encoding coiled-coil alpha-helical rod protein 1 (CCHCR1) is in a genomic locus that is associated with the development of psoriasis [11]. Ling and colleagues used indirect immunofluorescence to show that CCHCR1 localizes to P-bodies and pull-down studies to show that the protein co-immunoprecipitates with P-body components, including EDC4, XRN1, PATL1 and DCP1a [10]. To further investigate the interaction between CCHCR1 and EDC4 inside cells, NLS-EDC4 and CCHCR1-Myc were co-expressed in HEp-2 cells. In cells expressing both proteins, CCHCR1 co-localized with NLS-EDC4 in nuclear dots (Fig 5G–5I). Similarly, co-expression of GFP-NLS-EDC4(630–1437) and CCHCR1-Myc (S3G–S3I Fig) or GFP-NLS-EDC4(935–1437) and CCHCR1-Myc (Fig 5J–5L) resulted in co-localization of the proteins in nuclear dots, showing that the C-terminus of EDC4 is sufficient to mediate interaction with CCHCR1.

### Interactions between DDX6 and the mRNA processing body

DDX6 has important roles in repression of translation, decapping of mRNAs and host defense. Previous investigators observed that the C-terminal domain in DDX6 is sufficient for localization to P-bodies [23] and that this portion of DDX6 mediates interaction with PATL1 [22]. To further investigate interactions between DDX6 and components of the P-body, a plasmid encoding the C-terminus of DDX6 (amino acids 289–483) fused to mCh and the SV40 T antigen NLS was prepared (mCh-NLS-DDX6(289–483)). When expressed in HEp-2 cells, this protein localized diffusely throughout the nucleus (S2K and S2L Fig). When NLS-EDC4 was co-expressed with mCh-NLS-DDX6(289–483), NLS-EDC4 localized to nuclear dots, while mCh-NLS-DDX6(289–483) remained diffusely distributed throughout the nucleus (S3J–S3L Fig), suggesting that EDC4 does not directly interact with this portion of DDX6.

**Fig 6:**
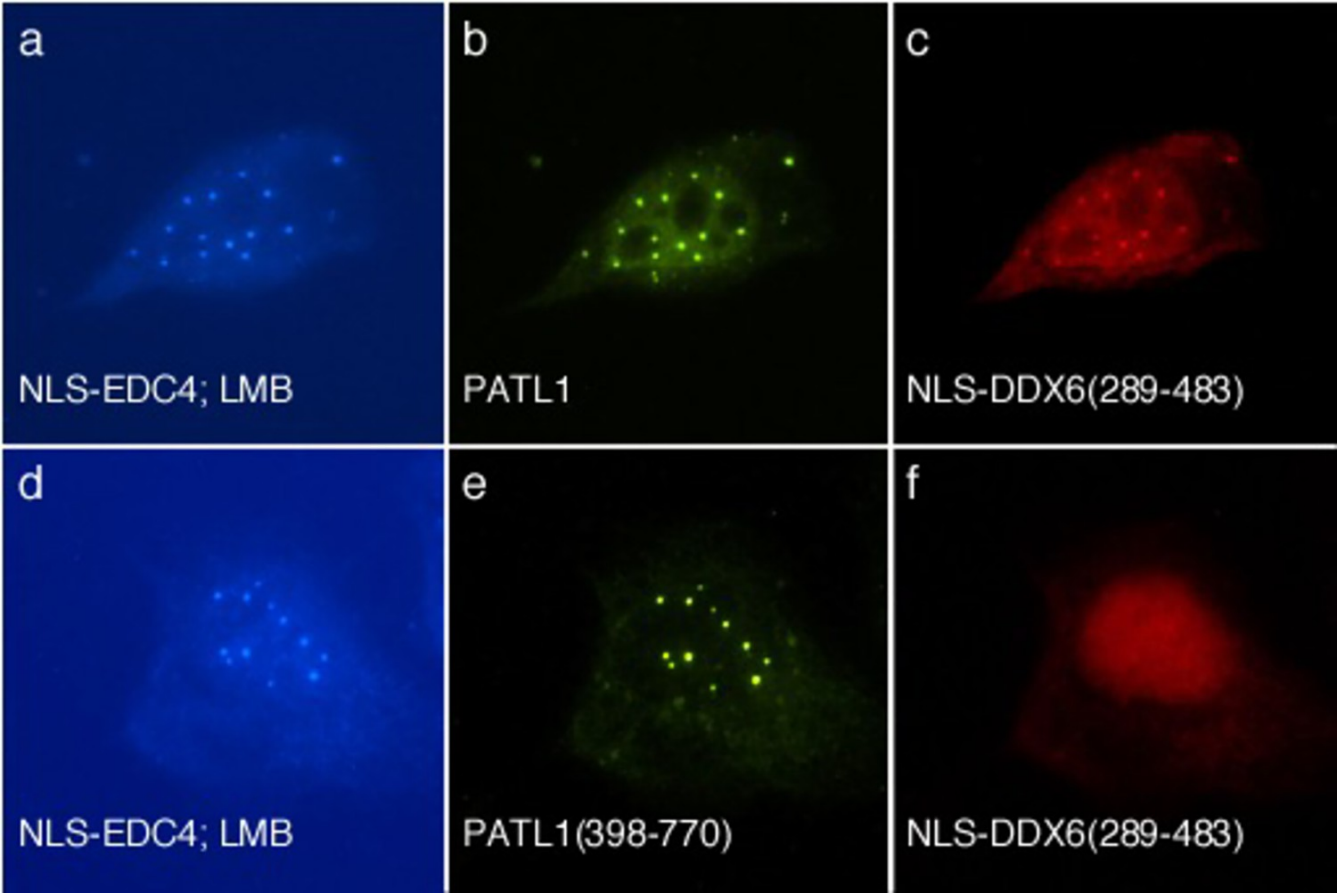
DDX6 interacts with PATL1. Co-expression of NLS-EDC4 (panel a), GFP-PATL1 (panel b) and monomeric cherry (mCh)-NLS-DDX6(289–483) (panel c) followed by treatment with leptomycin B (LMB) resulted in co-localization of the proteins in nuclear dots in 81+/-5% of cells expressing all three proteins. Faint, diffuse nuclear staining of mCh-NLS-DDX6(289–483) was also detected. Co-expression of NLS-EDC4, GFP-PATL1(398–770) and mCh-NLS-DDX6(289–483 followed by treatment with LMB resulted in localization of NLS-EDC4 (panel d) and GFP-PATL1(398–770) (panel e) in nuclear dots. However, in cells expressing all three proteins, mCh-NLS-DDX6(289–483) was not detected in nuclear dots (panel f). Human serum containing anti-EDC4 antibodies and coumarin-conjugated donkey anti-human IgG antiserum were used to detect NLS-EDC4 in a and d. Mouse monoclonal anti-GFP antibody detected GFP-PATL1 (panel b) and GFP-PATL1(398–770) (panel e). Rabbit anti-mCh antiserum detected mCh-NLS-DDX6(289–483) in c and f.

To investigate whether DDX6 interacts with PATL1 inside cells, as was previously reported [22], HEp-2 cells were transfected with plasmids encoding NLS-EDC4, GFP-PATL1 and mCh-NLS-DDX6(289–483). Cells were subsequently treated with LMB for two hours to inhibit nuclear export and were stained with human serum containing antibodies directed against EDC4, mouse anti-GFP antibodies and rabbit anti-mCh antiserum. In cells expressing NLS-EDC4, GFP-PATL1 and mCh-NLS-DDX6(289–483), all three proteins localized in nuclear dots (Fig 6A–6C). Taken together, the results confirm that DDX6 interacts with PATL1.

Ozgur and Stoecklin used co-immunoprecipitation to show that the N-terminus of PATL1 was required to mediate interaction with DDX6 [22]. To confirm this observation, HEp-2 cells were transfected with plasmids encoding NLS-EDC4, GFP-PATL1(398–770), and mCh-NLS-DDX6(289–483) and were then treated with LMB. Under these conditions, PATL1(398–770) co-localized with NLS-EDC4 in nuclear dots. However, mCh-DDX6(289–483) was detected diffusely throughout the nucleus (Fig 6D–6F). The results confirm that the N-terminus of PATL1 is required to mediate interaction with DDX6.

#### Depletion of LSm14a or DDX6 impairs the ability of full-length EDC4, but not EDC4(630–1437), to form cytoplasmic dots

**Fig 7:**
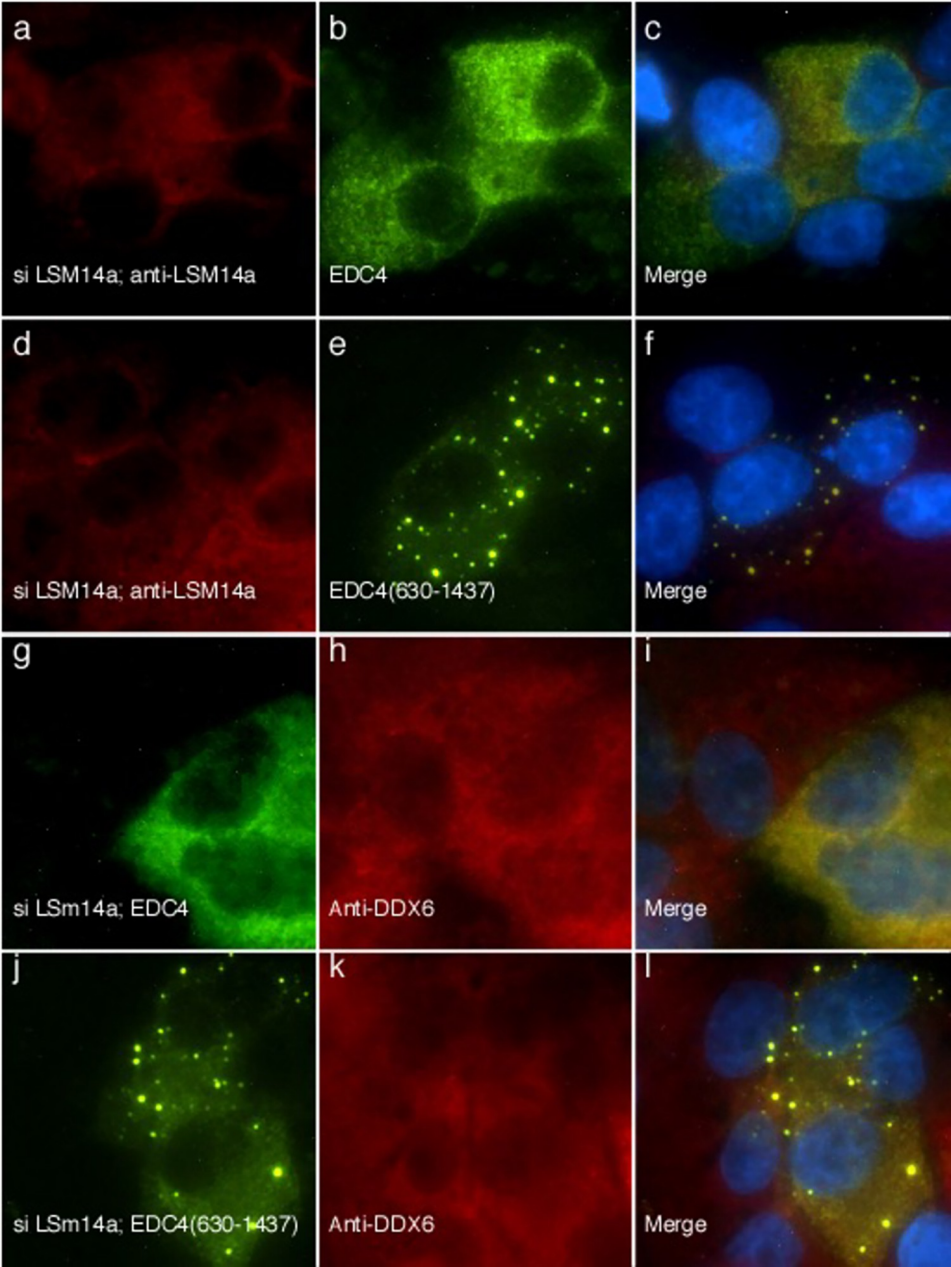
After depletion of LSm14a and loss of endogenous P-bodies, GFP-EDC4(630–1437), but not GFP-EDC4, localizes to cytoplasmic dots. siRNA directed against LSm14a was transfected into HEp-2 cells, followed 24 hours later by transfection of GFP-EDC4 or GFP-EDC4(630–1437). LSm14a was not detected in siLSm14a-treated cells (panel a). GFP-EDC4 (panels b, c) was unable to localize to P-bodies and was distributed throughout the cytoplasm of transfected cells. In cells depleted of LSm14a (panel d) and expressing GFP-EDC4(630–1437) (panels e, f), GFP-EDC4(630–1437) was detected in cytoplasmic dots that were indistinguishable from P-bodies at the level of UV light microscopy. **After disruption of endogenous P-bodies, neither full-length EDC4 nor EDC4(630–1437) was able to recruit DDX6 to cytoplasmic dots.** After depletion of LSm14a using siRNA, GFP-EDC4 (panel g) did not form cytoplasmic dots and was unable to recruit endogenous DDX6 (panel h) to cytoplasmic dots. After depletion of LSm14a, GFP-ED4(630–1437) (panel j) was able to form cytoplasmic dots, but was unable to recruit endogenous DDX6 (panels k, l) to these structures. Rabbit antiserum was used to confirm the absence of LSm14a in a and d. Mouse anti-GFP antibodies detected GFP-EDC4 (b, g) and GFP-EDC4(630–1437) (e and j). Rabbit anti-DDX6 antiserum was used to detect endogenous DDX6 in h and k. Merge of panels a and b, d and e, g and h, and j and k is shown in c, f, i, and l, respectively. DAPI staining (blue) in c, f, i and l indicate the location of nuclei.

Depletion of LSm14a or DDX6 results in loss of detectable P-bodies, suggesting that these proteins may be integral, structural components of P-bodies [19, 20, 23, 24]. To further investigate the role of LSm14a in the formation of P-bodies, HEp-2 cells were depleted of LSm14a using siRNA and subsequently transfected with GFP-EDC4. As expected, depletion of LSm14a resulted in loss of endogenous P-bodies and transfected GFP-EDC4 was unable to localize to cytoplasmic dots (Fig 7A–7C). However, in cells depleted of LSm14a but transfected with GFP-EDC4(630–1437), GFP-EDC4(630–1437) formed cytoplasmic dots that were indistinguishable from P-bodies at the level of UV microscopy, despite the absence of LSm14a (Fig 7D–7F). The results suggest that LSm14a is not structurally required for the stability of P-bodies, but rather that the lack of interaction between LSm14a and the N-terminus of EDC4 results in dissolution of these structures. In the absence of the N-terminus of EDC4, LSm14a was no longer required to maintain the stability of these cytoplasmic dots.

#### In the absence of endogenous P-bodies, EDC4(630–1437) is unable to recruit LSm14a or DDX6 to cytoplasmic dots

In cells depleted of LSm14a and subsequently transfected with GFP-EDC4, neither GFP-EDC4 nor endogenous DDX6 localized to P-bodies (Fig 7G–7I). After depletion of LSm14a and transfection with GFP-EDC4(630–1437), GFP-EDC4(630–1437) formed cytoplasmic dots, but endogenous DDX6 was unable to localize to these structures (Fig 7J–7L). Similar results were observed when cells were depleted of DDX6: GFP-EDC4(630–1437), but not GFP-EDC4, was able to form cytoplasmic dots and endogenous LSm14a was unable to join GFP-EDC4(630–1437) in these structures (S5 Fig). The results suggest that LSm14a and DDX6 are functionally, but not structurally, required for EDC4 to form cytoplasmic dots. In addition, the N-terminus of EDC4 is required to recruit LSm14a and DDX6 to P-bodies.

#### In the absence of endogenous P-bodies, EDC4(630–1437) can recruit DCP1a, EDC3 and CCHCR1 to cytoplasmic dots

**Fig 8:**
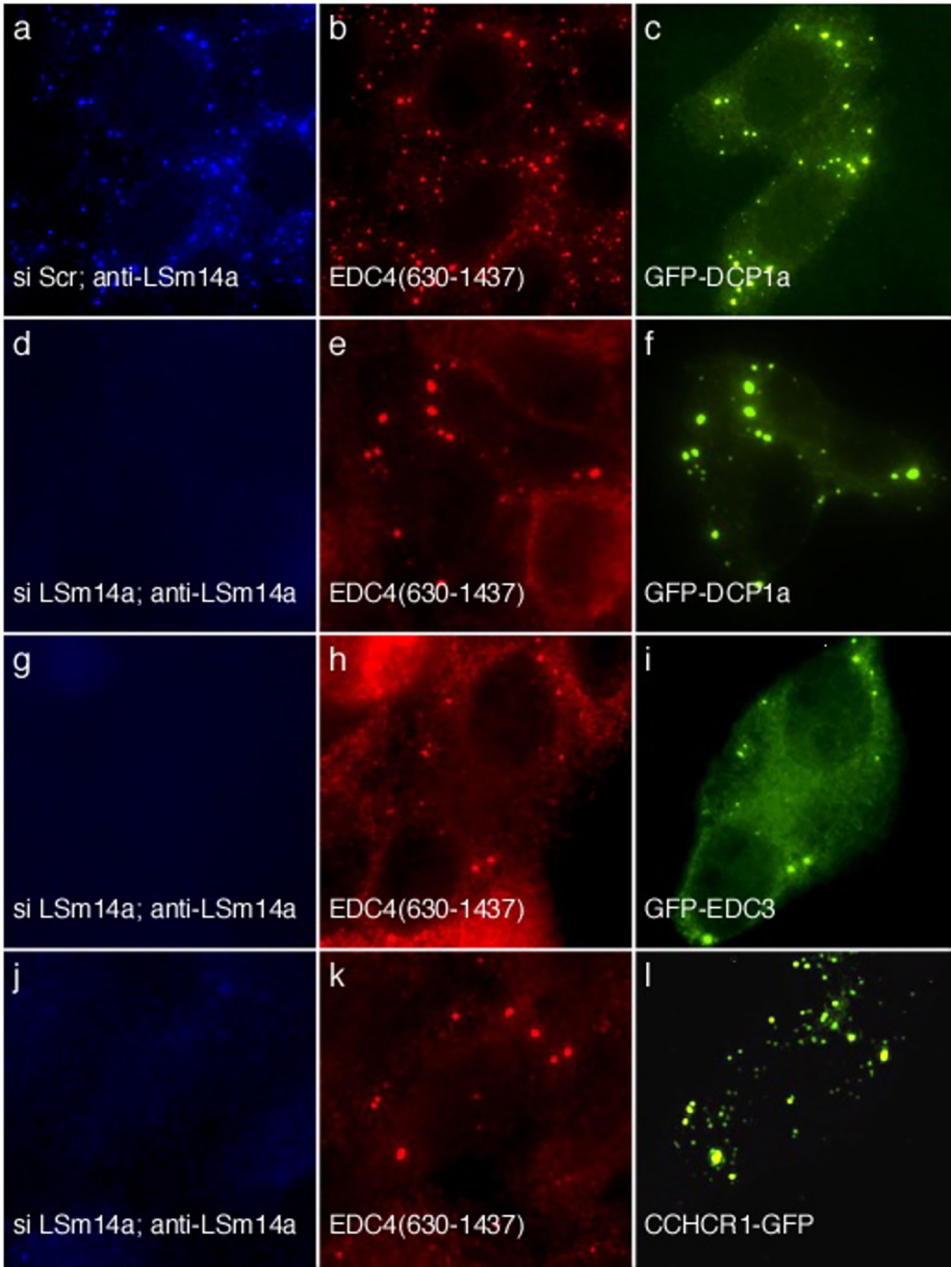
EDC4(630–1437) can recruit DCP1a, EDC3 or CCHCR1 to cytoplasmic dots despite depletion of LSm14a and loss of endogenous P-bodes. After treatment with scrambled siRNA, LSm14a (panel a), EDC4(630–1437) (panel b) and GFP-DCP1a (panel c) all localized in cytoplasmic dots. siRNA directed against LSm14a resulting in depletion of LSM14a from HEp-2 cells (panel d), but EDC4(630–1437) (panel e) and GFP-DCP1a (panel f) localized to cytoplasmic dots. Depletion of LSm14a (panels g, j) did not affect localization of EDC4(630–1437) (panels h, k) and EDC3 (panel i) or CCHCR1 (panel l) to cytoplasmic dots. Rabbit antiserum was used to detect LSm14a in panel a and to confirm the absence of LSm14a and endogenous P-bodies in d, g, and j. Human serum reacted with EDC4(630–1437) in b, e, h and k. Mouse anti-GFP antibody detected GFP-DCP1a in c and f, GFP-EDC3 in i, and CCHCR1-GFP in l.

To determine whether EDC4(630–1437) retains the ability to recruit other P-body components to cytoplasmic dots, despite the absence of endogenous P-bodies and loss of the N-terminal domain, cells were treated with siRNA directed against LSm14a and then transfected with EDC4(630–1437) and GFP-DCP1a, GFP-EDC3, or CCHCR1-GFP. Control cells were treated with a scrambled siRNA (scr siRNA) and were transfected with EDC4(630–1437) and GFP-DCP1a. In control cells, EDC4 and DCP1a localized with LSm14a in P-bodies (Fig 8A–8C). In cells treated with siRNA directed against LSm14a and transfected with EDC4(630–1437) and GFP-DCP1a, GFP-DCP1a co-localized with EDC4(630–1437), despite the absence of LSm14a (Fig 8D–8F). Similarly, GFP-EDC3 (Fig 8G–8I) and CCHCR1-GFP (Fig 8J–8L) co-localized with EDC4(630–1437) in cytoplasmic dots, despite the absence of LSm14a. Taken together, the results suggest that LSm14a is functionally, but not structurally, required for EDC4 to recruit P-body components DCP1a, EDC3 and CCHCR1 to cytoplasmic dots.

## Discussion

In previous studies, we used a fluorescence-based, intracellular two-hybrid assay to investigate interactions between protein components of the mammalian mRNA P-body and demonstrated interactions between EDC4 and DCP2, DCP1a, and MARF1 as well as EDC4 dimerization [8, 25]. In the present study, we used the two-hybrid assay to show that EDC4 interacts with LSm14a, PATL1, XRN1, NBDY and CCHCR1 inside the cell. The full-length protein, including the N-terminal WD40 domain, was required to mediate interaction between EDC4 and the first four of these P-body components. In addition, interaction between EDC4 and DDX6 was bridged by PATL1, and the N-terminus of PATL1 was required for interaction with DDX6. The C-terminal, alpha helix-rich domain in EDC4 was sufficient for interactions between EDC4 and CCHCR1 and DCP1a.

As shown in previous studies, depletion of DDX6 or LSm14a resulted in loss of endogenous P-bodies [19, 20, 23] and overexpression of GFP-EDC4 was unable to restore these structures. In contrast, even after depletion of DDX6 or LSm14a, EDC4 lacking the N-terminus of the protein was able to localize to cytoplasmic dots that were indistinguishable from P-bodies at the level of UV light microscopy. In the absence of DDX6 and endogenous P-bodies, EDC4(630–1437) was able to recruit DCP1a, CCHCR1 and EDC3, but not LSm14a, to cytoplasmic dots. These studies, involving reconstitution of cytoplasmic dots in cells that lack endogenous P-bodies, confirm the results of the mammalian two-hybrid assay: The N-terminus of EDC4 is required for interaction with LSm14a, but is dispensable for interaction between EDC4 and DCP1a, CCHCR1 and EDC3.

### P-body components in the nucleus

When forced to enter the nucleus, because of fusion to an exogenous NLS, NLS-EDC4 localized to Cajal bodies. Without an exogenous NLS, EDC4 was not detected in the nucleus, even after inhibition of nuclear export for 5 hours using LMB [30]. However, in a previous study, we showed that EDC4 has a functional NLS [26]. The presence of this NLS and the observation that EDC4 fused to an exogenous NLS specifically localizes to Cajal bodies, suggest that under physiological conditions, a small fraction of cellular EDC4 may be present in the nucleus. The protein or proteins that mediate localization of EDC4 to Cajal bodies is/are unknown.

PATL1 is a protein that shuttles between the nucleus and cytoplasm. In the nucleus, PATL1 participates in mRNA splicing, while in the cytoplasm, PATL1 inhibits mRNA translation and enhances mRNA decapping (reviewed in [29]). Marnef and colleagues showed that a small fraction of cellular PATL1 has a nuclear distribution that is similar to that of NLS-EDC4: localization to Cajal bodies and adjacent to a subset of PML nuclear bodies [29, 30]. In this study, NLS-EDC4 appeared to determine the location of nuclear PATL1, because when NLS-EDC4 and PATL1 were co-expressed in cells that were subsequently treated with LMB, PATL1 was only seen in nuclear dots; homogeneous and finely speckled nuclear staining patterns were no longer visible. Whether a small fraction of endogenous EDC4 recruits PATL1 to Cajal bodies is unknown.

### Interactions between EDC4 and DDX6, LSm14a, and XRN1

DDX6 is an RNA helicase that is required for P-body formation. Mammalian DDX6 was reported to be recruited to P-bodies by both PATL1 and EDC3. Ozgur and Stoecklin used co-immunoprecipitation to show that the C-terminal domain of DDX6 interacts with the N-terminal portion of PATL1 [22]. Tritschler and colleagues used bacterially-expressed human EDC3 (amino acids 192–228) to purify the C-terminal portion of DDX6 (amino acids 296–472) and determined the crystal structure of the interacting protein fragments [34]. In this study, we used the mammalian two-hybrid assay to confirm the interaction between PATL1 and DDX. However, we were unable to confirm interaction between EDC3 and DDX6: In cells that lacked LSm14a and endogenous P-bodies, EDC4(630–1437) was able to recruit DCP1a and EDC3, but not DDX6, to cytoplasmic dots. The results suggest that in mammalian cells, interaction between EDC3 and DDX6 is not a major pathway for localization of DDX6 to P-bodies.

The mechanism by which LSm14a localizes to P-bodies was previously unknown. Brandmann and colleagues showed that a bipartite binding motif in LSM14a (FDF and TFG domains) mediates interaction with the C-terminal portion of DDX6 and suggested that the interaction between LSm14a and DDX6 allows a third domain in LSm14a (FFD motif) to recruit EDC4 to P-bodies [35]. In this study, we showed that NLS-EDC4 was able to change the nuclear distribution of NLS-LSm14a from diffuse homogenous to nuclear dots and that the N-terminal, WD40-containing portion of NLS-EDC4 was required to alter the nuclear location of NLS-LSm14a. In the absence of endogenous P-bodies, EDC4(630–1437) retained the ability to form cytoplasmic dots and recruit DCP1a, CCHCR1 and EDC3, but not LSm14a, to these structures. Taken together, the results suggest that EDC4 determines the cellular location of LSm14a and that the N-terminus of EDC4 is required for the interaction between the two proteins.

XRN1 mediates the 5’ to 3’ degradation of uncapped mRNA. Braun and colleagues showed that XRN1 amino acids 1174–1706 co-precipitated with EDC4 amino acids 974–1401 [31]. In this study, using both the two-hybrid assay and co-immunoprecipitation, we found that full-length EDC4, but not EDC4 lacking the N-terminal domain, was required to mediate interaction with the C-terminal portion of XRN1. The reason for the conflicting results in uncertain.

### Interactions between the C-terminus of EDC4 and DCP1a and CCHCR1

In yeast, the decapping activator DCP1a interacts directly with the decapping enzyme (DCP2). In mammalian cells, EDC4 acts as a scaffold to assemble the DCP1a/DCP2 complex and the direct interaction between DCP1 and DCP2 is weak [33]. We and others showed that DCP2 interacts with the C-terminal portion of EDC4 [25, 36]. The mechanism by which DCP1a interacts with EDC4 is uncertain. Chang and colleagues showed that EDC4 amino acids 1–538, but not 974–1401, were sufficient to immunoprecipitate DCP1a [33]. In this study, using the two-hybrid assay, we observed that the N-terminus of EDC4 is not required to mediate interaction between the two proteins. DCP2 is unlikely to bridge the interaction between EDC4(630–1437) and DCP1a because the level of endogenous DCP2 is relatively low compared to the transiently expressed EDC4(630–1437) and DCP1a. Also, as noted by Chang and colleagues, direct interaction between DCP1a and DCP2 in mammalian cells is weak. In addition, in this study, we showed that in the absence of endogenous P-bodies, EDC4(630–1437) was able to recruit GFP-DCP1a to cytoplasmic dots. Taken together, the results show that the N-terminus is not required for interaction between EDC4 and DCP1a. The results do not exclude the possibility of a second interaction between DCP1a and the N-terminus of EDC4.

The gene encoding CCHCR1 maps to a genetic locus that is associated with an increased risk of psoriasis. Although the function of this protein is unknown, CCHCR1 may have a role in the regulation of keratinocyte proliferation [11]. The protein may also participate in the pathogenesis of human papillomavirus Type 16 infection [9]. Ling and colleagues showed that CCHCR1 localizes to P-bodies and co-immunoprecipitates with EDC4, XRN1, PATL1 and DCP1a [10]. In this study, NLS-EDC4 recruited CCHCR1 to nuclear dots and the C-terminus of EDC4 was sufficient to mediate this interaction. CCHCR1 joins DCP2, DCP1a and EDC4 itself as proteins that interact with the alpha helix-rich C-terminal portion of EDC4.

A potential limitation of the present study is that we have not demonstrated a direct interaction between EDC4 and P-body components LSm14a, PATL1, XRN1, DCP1a and CCHCR1. Because HEp-2 cells have endogenous P-body proteins, it is possible that each “prey” P-body component co-localized in nuclear dots with the “bait” (NLS-EDC4 or fragments of EDC4) because of the presence of one or more “bridging” proteins. However, this possibility seems unlikely, because each of the endogenous P-body components has a strong nuclear export sequence and is present in low concentration relative to over-expressed NLS-EDC4. For example, after overexpression of NLS-EDC4 in HEp-2 cells, we were unable to detect endogenous LSm14a or DDX6 in nuclear dots (S6 Fig), suggesting that neither of these proteins is able to bridge an interaction between NLS-EDC4 and other, over-expressed P-body components. In addition, in a previous study [8], we showed that overexpressed EDC3 was unable to co-localize with NLS-EDC4 unless the bridging protein (DCP1a) was simultaneously overexpressed in HEp-2 cells, again suggesting that endogenous DCP1a is unable to bridge the interaction between NLS-EDC4 and EDC. Endogenous PATL1 and XRN1 are also unlikely to serve as links between NLS-EDC4 and other P-body components: In this study, we found that overexpressed PATL1 did not co-localize with NLS-EDC4 unless the nuclear export sequence was removed; overexpressed XRN1 did not co-localize with NLS-EDC4 unless an exogenous NLS was fused to the protein. Taken together, these considerations suggest that endogenous P-body components are unable to bridge an interaction between NLS-EDC4 and an over-expressed, prey P-body component and that the two-hybrid assay is able to detect direct protein interactions that occur within the cell.

## Conclusion

**Fig 9:**
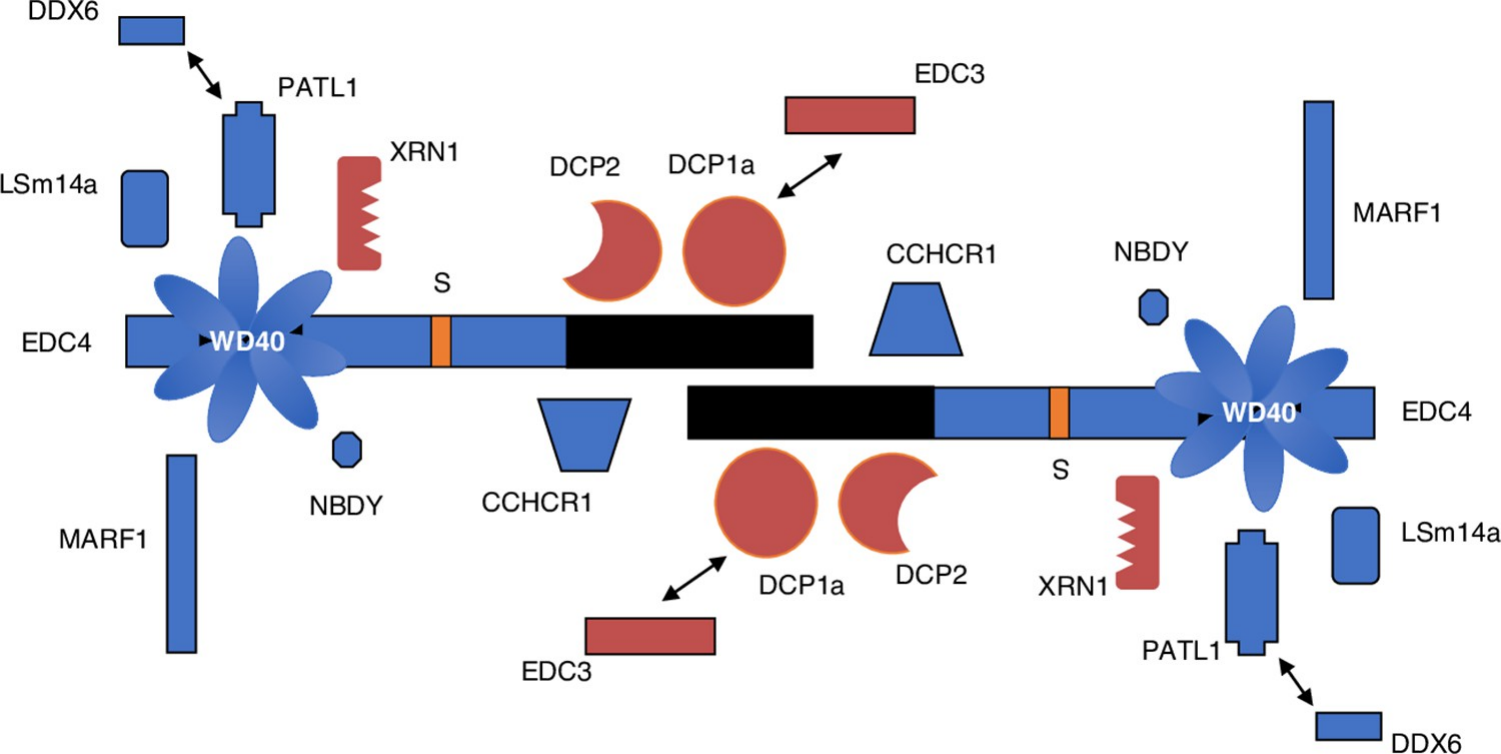
Model of the mammalian mRNA P-body. Interactions between EDC4 and LSm14a, PATL1, XRN1, MARF1, NBDY and DDX6 (via PATL1) require the N-terminal, WD40-containing domain in EDC4. The C-terminus of EDC4 is sufficient to mediate interaction between EDC4 and DCP2, DCP1a, CCHCR1 and EDC3 (via DCP1a). RNA binding proteins DDX6, LSm14a, PATL1, and MARF1 may be present on the “outer surface” of the P-body, while the decapping protein (DCP2) and enhancers of decapping may be “buried”, and perhaps inactive, inside P-bodies. Phosphorylation of NBDY or depletion of LSm14a or DDX6 results in disruption of P-bodies.

The results of this study permit the development of a new model of the structure and regulation of mammalian P-bodies (Fig 9): The decapping enzyme (DCP2) and enhancers of decapping (DCP1a, EDC3) interact with the C-terminal portion of EDC4 (EDC3 via interaction with DCP1a). RNA binding proteins PATL1, DDX6, and LSm14a require the N-terminal, WD40-containing domain in EDC4 to mediate interaction. EDC4 is shown as forming an anti-parallel dimer because of the structure observed by Jinek and colleagues, who crystalized the C-terminus of EDC4 [27]. The model suggests that the decapping enzyme and decapping activators may be “hidden” within the center of the P-body, while components that bind and store mRNA are present on the outside of the structure. By interacting with the N-terminus of EDC4, phosphorylated NBDY may cause a conformational change that interferes with the ability of EDC4 to interact with other P-body components at both the N- and C-terminal portions of the protein. Similarly, depletion of DDX6 or LSm14a, as might occur during the course of a viral infection [37], also results in P-body disassembly. In the presence of phosphorylated NBDY or the absence of DDX6 or LSm14a, P-bodies may release mRNAs which are translated and may mediate a coordinated response to internal or external stimuli. Of note, dissolution of P-bodies might also permit activation of the internal decapping proteins, resulting in enhanced degradation of mRNAs. The mechanisms that determine whether P-bodies provide mRNAs for translation or enzymes to facilitate mRNA degradation remain to be explored.

## Supporting information

S1 File Reagents and resources. (DOCX)

S1 Fig Schematic of P-body proteins and fragments of proteins that were used in this study. The black box at the N-terminus of EDC4, EDC4(630–1437), EDC4(935–1437), LSm14a, XRN1(1232–1706), DCP1a and DDX6(289–483) indicates the location of the exogenous nuclear localization sequence.
(TIF)

S2 Fig Effect of an exogenous nuclear localization sequence (NLS) on the cellular location of P-body components. Expression of NLS-EDC4 (panels a, b) and GFP-NLS-EDC4 (panels c, d) in HEp-2 cells resulted in localization of the protein to nuclear dots. In contrast, diffuse nuclear staining was seen after transfection of plasmids encoding NLS fused to DCP1a (panels e, f), LSm14a (panels g, h) or DDX6 amino acids (289–483) (panels k and l). Addition of a NLS to the N-terminus of XRN1 amino acids 1232–1706 had no effect on the distribution of the protein fragment, which localized to cytoplasmic dots (panels I, j). Human serum was used to detect EDC4 in panel a. Mouse anti-GFP antibody detected GFP-NLS-EDC4 in panel c. Rabbit antiserum was used to detect NLS-DCP1a (panel e) or NLS-LSm14a (panel g). Rabbit anti-monomeric cherry (mCh) antiserum was used to detect mCh-NLS-XRN1(1232–1706) and mCh-NLS-DDX6(289–483) in i and k, respectively. DAPI staining in b, d, f, h, j and l indicates the location of nuclei in the preceding panels.
(TIF)

S3 Fig When expressed in HEp-2 cells, GFP-NLS-EDC4 (panel a) localized to Cajal bodies (panels b, c). Co-expression of NLS-EDC4 (panel d) and mCh-XRN1(1232–1706) (panel e) resulted in localization of NLS-EDC4 to nuclear dots, while mCh-XRN1(1232–1706) localized to cytoplasmic dots. Expression of GFP-NLS-EDC4(630–1437) (panel g) and CCHCR1-Myc (panel h) in HEp-2 cells resulted in localization of both proteins to nuclear dots. Co-expression of NLS-EDC4 and mCh-NLS-DDX6(289–483) in HEp-2 cells resulted in localization of NLS-EDC4 to nuclear dots (panel j), while mCh-NLS-DDX6(289–483) was distributed diffusely throughout the nucleus (panel k). Mouse monoclonal anti-GFP antibody was used to detect GFP-NLS-EDC4 (panel a) and GFP-NLS-EDC4(630–1437) (panel g). Human serum containing anti-Cajal antibodies was used in panel b. Human serum containing anti-EDC4 antibodies was used to detect NLS-EDC4 in (panels d and j). Rabbit anti-mCh antiserum was used to detect mCh-XRN1(1232–1706) and mCh-NLS-DDC6(289–483) (panels e and k). Rabbit anti-Myc antiserum was used to detect CCHCR1-Myc in panel h. Merge of panels a and b, d and e, g and h, j and k is shown in c, f, i, and l, respectively. DAPI staining in f and i indicate the location of nuclei.
(TIF)

S4 Fig Co-immunoprecipitation was used to show that the N-terminus of EDC4 is required for interaction with XRN1(1232–1706). HEK293 cells were transfected with mCh-XRN1(1232–1706) alone (lane 1), mCh-XRN1(1232–1706) and GFP-EDC4(630–1437) (lane 2), or mCh-XRN1(1232–1706) and GFP-EDC4 (lane 3). Panel a shows the amount of mCh-XRN1(1232–1706) in 2% of the input protein extract. Panels b and c show that GFP-EDC4 (lane 3), but not the magnetic beads alone (lane 1) or GFP-EDC4(630–1437) (lane 2) immunoprecipitated mCh-XRN1(1232–1706). mCh-XRN1(1232–1706) was detected using rabbit anti-mCh antiserum. GFP fusion proteins were detected using mouse anti-GFP antibody.
(TIF)

S5 Fig After depletion of DDX6 and loss of endogenous P bodies, EDC4(630–1437), but not full-length EDC4, was able to localize to cytoplasmic dots. Neither full-length EDC4 nor EDC4(630–1437) was able to recruit endogenous LSm14a to cytoplasmic dots. After depletion of DDX6 using siRNA, neither GFP-EDC4 (panel a) nor endogenous LSm14a (panel b) localized to cytoplasmic dots. After depletion of DDX6, GFP-EDC4(630–1437), but not endogenous LSm14a localized to cytoplasmic dots. Mouse anti-GFP antibody was used to detect GFP-EDC4 (panel a) and GFP-EDC4(630–1437) (panel d). Rabbit anti-LSm14a antiserum was to stain for LSm14a in panels b and e. Merge of panels a and b, and d and e is shown in c and f respectively. DAPI staining in c and f indicate the location of nuclei.
(TIF)

S6 Fig Endogenous LSm14a and DDX6 did not co-localize with NLS-EDC4 in nuclear dots. After expression of a plasmid encoding NLS-EDC4 in HEp-2 cells, human serum containing anti-EDC4 antibodies detected EDC4 in both nuclear and cytoplasmic dots (panels a and d). Endogenous LSm14a (panel b) and DDX6 (panel e) were only detected in cytoplasmic dots. Rabbit anti-LSm14a and anti-DDX6 antisera were used to detect the corresponding proteins. Merge of panels a and b, and c and e, is shown in c and f, respectively. DAPI staining in c and f indicate the location of nuclei in the preceding panels.
(TIF)
