# Supplementary material for: Assembly and regulation of the mammalian mRNA processing body
Source: PLoS One. 2023 Mar 6;18(3):e0282496. doi: 10.1371/journal.pone.0282496 (PMC9987799; doi:10.1371/journal.pone.0282496)
Supplement: S1 File — (DOCX) [file pone.0282496.s001.docx]

| **Supporting Information**  **Table 1**  **Reagent or resource** |  |  |
| --- | --- | --- |
| **Plasmids** | **Source** | **Identifier** |
|  |  |  |
| **Epitopes** |  |  |
| pEGFP | Clontech | cat #6083-1 |
| pEGFP-NLS | Ref (1) |  |
| mCherry2-C1 | Addgene | cat #54563 |
| mCherry2-C1-NLS | oligonucleotides below |  |
| pCMV-FLAG | Agilent technologies | cat# 211172 |
| pCMV-Myc | Agilent technologies | cat #211173 |
|  |  |  |
| **EDC4** |  |  |
| GFP-NLS-EDC4(1-1437) | Ref (1) |  |
| pCDNA3-NLS-EDC4(1-1437) | Ref (1) |  |
| GFP-NLS-EDC4(630-1437) | restriction enzymes |  |
| GFP-NLS-EDC4(1095-1437) | Ref (1) |  |
| GFP-EDC4(1-1437) | Ref (1) |  |
| pCMV-EDC4(630-1437) | restriction enzymes |  |
|  |  |  |
| **PATL1** |  |  |
| GFP-PATL1 | Genecopoeia | cat# EX-H2755-M29 |
| GFP-PATL1(398-770) | restriction enzymes |  |
| Myc-PATL1 | Genecopoeia | cat# EX-H2755-M43 |
| Myc-PATL1(398-770) | restriction enzymes |  |
|  |  |  |
| **LSm14a** |  |  |
| GFP-LSm14a | Ref (2) |  |
| pCDNA3-NLS-LSm14a | restriction enzymes |  |
| GFP-NLS-LSm14a | restriction enzymes |  |
|  |  |  |
| **XRN1** |  |  |
| pLNHA-Cl-HsXRN1 | Addgene | cat# Z753-5276 |
| mCh-XRN1(1232-1706) | restriction enzymes |  |
| mCh-NLS-XRN1(1232-1706) | restriction enzymes |  |
|  |  |  |
|  |  |  |
| **NBDY** |  |  |
| NBDY | HEp-2 cell cDNA, oligonucleotides below |  |
| mCh-NBDY | restriction enzymes |  |
|  |  |  |
| **DCP1a** |  |  |
| GFP-DCP1A | Genecopoeia | cat# Ex-V1649-M29 |
| pCDNA3-NLS-DCP1a | restriction enzymes |  |
|  |  |  |
|  |  |  |
| **CCHCR1** |  |  |
| CCHCR1-myc | Genecopoeia | cat# Ex-Z29219-M10 |
| CCHCR1-GFP | Genecopoeia | cat# Ex-Z29219-M03 |
|  |  |  |
| **DDX6** |  |  |
| pcDNA-DDX6 | J. Lykke-Andersen (Univ CA, San Diego) |  |
| mCh-NLS-DDX6(289-483) | restriction enzymes |  |
| GFP-DDX6(289-483) | restriction enzymes |  |
|  |  |  |
| **EDC3** |  |  |
| GFP-EDC3 | Genecopoeia | cat# Ex-Z6694-98 |
|  |  |  |
| **PCR oligonucleoties** |  |  |
| NBDY Forward | 5'-GGAGAAAACTGACGACCCGTTTCTGT-3' | |
| NBDY Reverse | 5'-TCTCTACTTCTCCGGAGGAGGAGGG-3' |  |
|  |  |  |
| **Linker oligonucleotides** |  |  |
| NLS forward | 5'-GATCCCCTAAGAAGAAGCGTAAGGTCAGA  TCTGCCGAATCCTCC-3' | |
| NLS reverse | 5'-AATTGGAGGATTCGGCAGATCTGACCTTAC  GCTTCTTCTTAGGG-3' | |
|  |  |  |
|  |  |  |
| **Antisera** |  |  |
| Human anti-EDC4 antibody | Ref (3) |  |
| Rabbit anti-Sp100 antiserum | Ref (4) |  |
| Rabbit anti-p80 coilin antiserum | Ed Chan, University of Florida |  |
| Human anti-p80 coilin antiserum | Clinical Immunology MGH | Reference serum |
| Mouse anti-GFP antibody | Roche Diagnostics | cat#-11814460001 |
| Rabbit anti-mCherry antibody | Invitrogen | cat# PAS-34974 |
| Rabbit anti-DCP1a antiserum | Abcam | cat# Ab183307 |
| Rabbit anti-DDX6 antiserum | Novus Biologicals | cat# NB200-191 |
| Rabbit anti-LSm14a antiserum | Abcam | cat# Ab229277 |
| Rabbit anti-Myc antiserum | Sigma | cat# C3956 |
|  |  |  |
| Secondary antisera | JacksonImmunoresearch |  |
| Donkey anti-human IgG |  |  |
| species-specific, FITC-conjugated |  | cat# 709-095-149 |
| species-specific, TRITC-conjugated |  | cat# 709-025-149 |
| species-specific, AMCA-conjugated |  | cat# 709-155-149 |
|  |  |  |
| Donkey anti-mouse IgG |  |  |
| species-specific, FITC-conjugated |  | cat# 715-095-150 |
| species-specific, TRITC-conjugated |  | cat# 715-025-150 |
|  |  |  |
| Donkey anti-rabbit IgG |  |  |
| species-specific, FITC-conjugated |  | cat# 711-095-152 |
| species-specific, TRITC-conjugated |  | cat# 711-025-152 |
| species-specific, AMCA-conjugated |  | cat# 711-155-152 |
|  |  |  |
| **Reagents** |  |  |
| Leptomycin B | Cell Signaling | cat# 9676S |
| Dharmafect | Dharmcon | cat# T2004-03 |
| Effectene | Qiagen | cat# 301425 |
| GFP-Trap magnetic agarose | Proteintech | cat# gtma |
| HEp-2 cells | American Type Cell Collection | cat# CCL-23 |
| HEK293 cells | American Type Call Collection | cat# CRL-1573 |

1. Bloch DB, Nobre RA, Bernstein GA, Yang WH. Identification and characterization of protein interactions in the mammalian mRNA processing body using a novel two-hybrid assay. Exp Cell Res. 2011;317(15):2183-99.

2. Yang WH, Yu JH, Gulick T, Bloch KD, Bloch DB. RNA-associated protein 55 (RAP55) localizes to mRNA processing bodies and stress granules. RNA. 2006;12(4):547-54.

3. Yang WH, Bloch DB. Probing the mRNA processing body using protein macroarrays and "autoantigenomics". RNA. 2007;13(5):704-12.

4. Bloch DB, Nakajima A, Gulick T, Chiche JD, Orth D, de La Monte SM, et al. Sp110 localizes to the PML-Sp100 nuclear body and may function as a nuclear hormone receptor transcriptional coactivator. Mol Cell Biol. 2000;20(16):6138-46.
