# Supplementary figures and images for: Assembly and regulation of the mammalian mRNA processing body
Source: PLoS One. 2023 Mar 6;18(3):e0282496. doi: 10.1371/journal.pone.0282496 (PMC9987799; doi:10.1371/journal.pone.0282496)

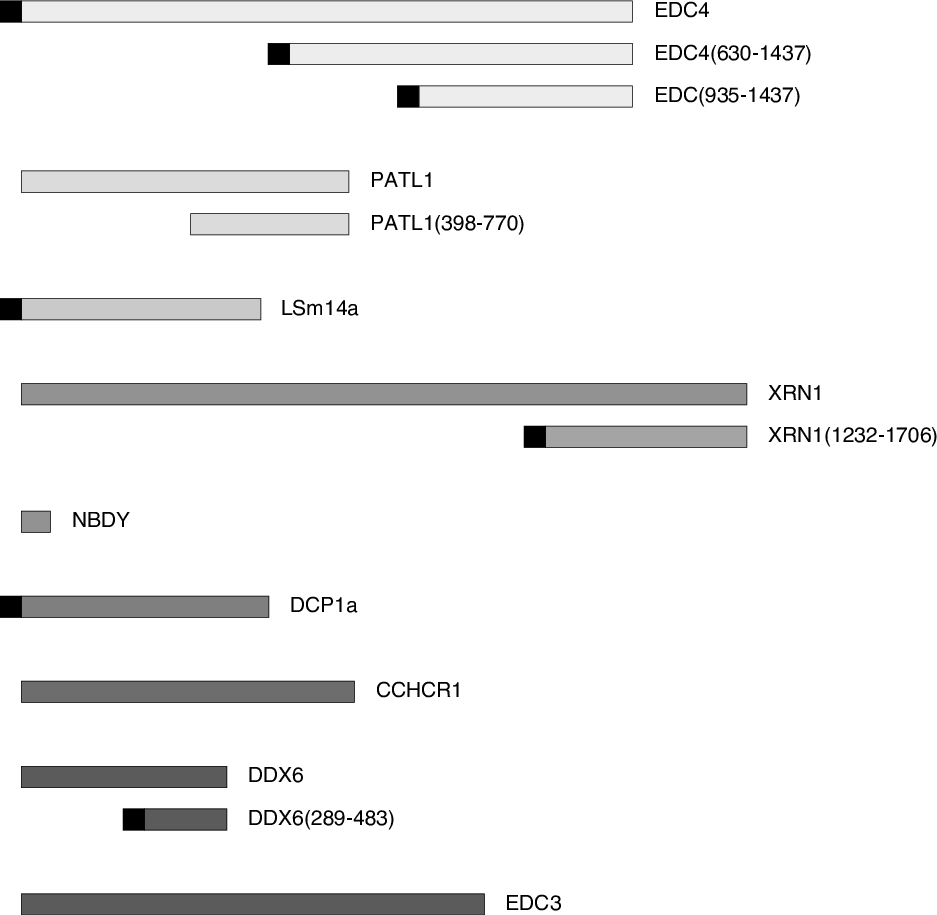

Supplement: S1 Fig — The black box at the N-terminus of EDC4, EDC4(630–1437), EDC4(935–1437), LSm14a, XRN1(1232–1706), DCP1a and DDX6(289–483) indicates the location of the exogenous nuclear localization sequence. (TIF) [file pone.0282496.s002.tif]

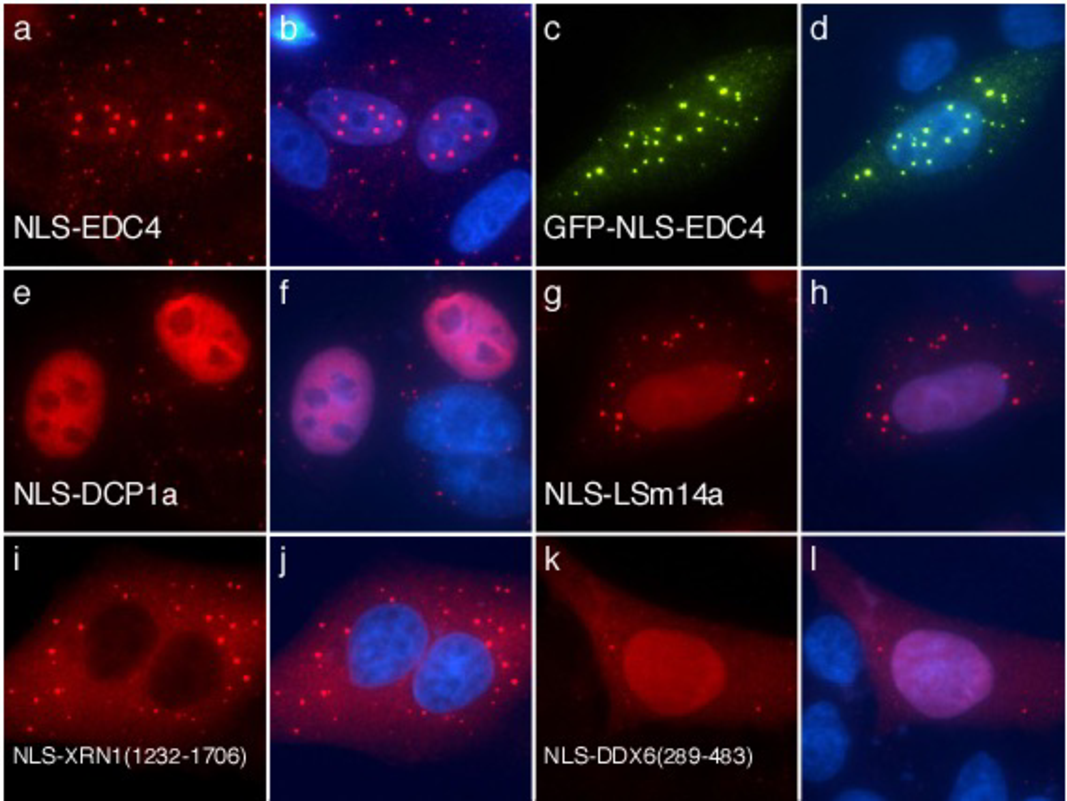

Supplement: S2 Fig — Expression of NLS-EDC4 (panels a, b) and GFP-NLS-EDC4 (panels c, d) in HEp-2 cells resulted in localization of the protein to nuclear dots. In contrast, diffuse nuclear staining was seen after transfection of plasmids encoding NLS fused to DCP1a (panels e, f), LSm14a (panels g, h) or DDX6 amino acids (289–483) (panels k and l). Addition of a NLS to the N-terminus of XRN1 amino acids 1232–1706 had no effect on the distribution of the protein fragment, which localized to cytoplasmic dots (panels I, j). Human serum was used to detect EDC4 in panel a. Mouse anti-GFP antibody detected GFP-NLS-EDC4 in panel c. Rabbit antiserum was used to detect NLS-DCP1a (panel e) or NLS-LSm14a (panel g). Rabbit anti-monomeric cherry (mCh) antiserum was used to detect mCh-NLS-XRN1(1232–1706) and mCh-NLS-DDX6(289–483) in i and k, respectively. DAPI staining in b, d, f, h, j and l indicates the location of nuclei in the preceding panels. (TIF) [file pone.0282496.s003.tif]

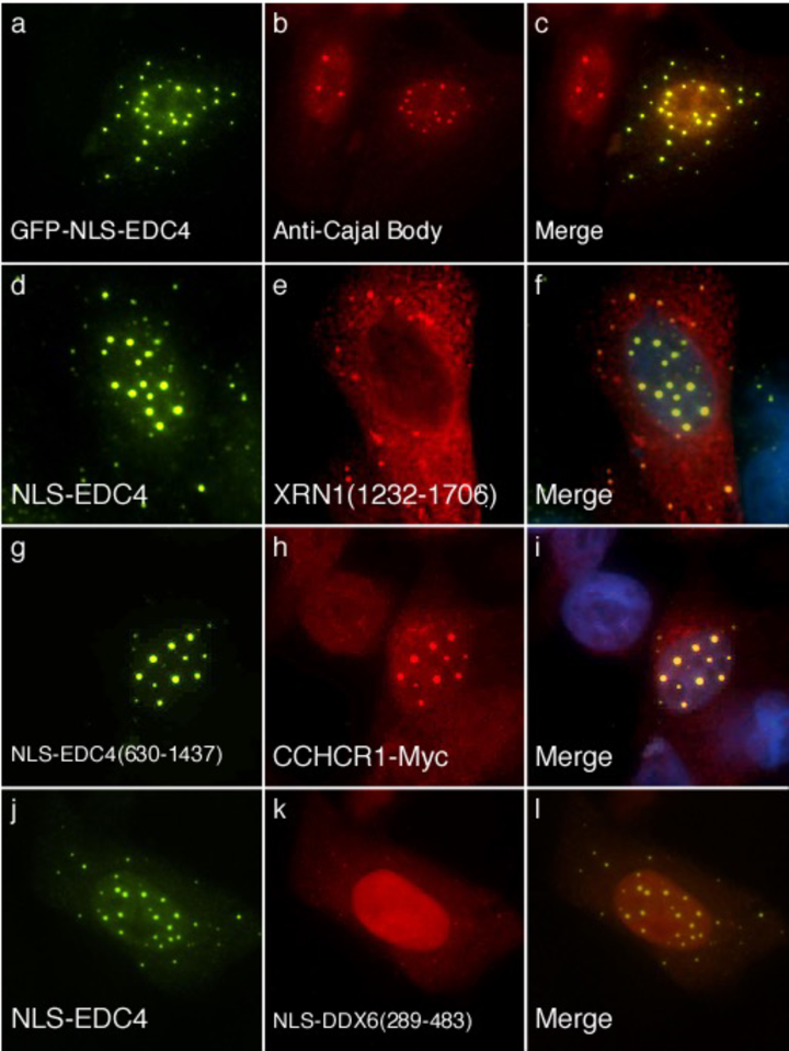

Supplement: S3 Fig — Co-expression of NLS-EDC4 (panel d) and mCh-XRN1(1232–1706) (panel e) resulted in localization of NLS-EDC4 to nuclear dots, while mCh-XRN1(1232–1706) localized to cytoplasmic dots. Expression of GFP-NLS-EDC4(630–1437) (panel g) and CCHCR1-Myc (panel h) in HEp-2 cells resulted in localization of both proteins to nuclear dots. Co-expression of NLS-EDC4 and mCh-NLS-DDX6(289–483) in HEp-2 cells resulted in localization of NLS-EDC4 to nuclear dots (panel j), while mCh-NLS-DDX6(289–483) was distributed diffusely throughout the nucleus (panel k). Mouse monoclonal anti-GFP antibody was used to detect GFP-NLS-EDC4 (panel a) and GFP-NLS-EDC4(630–1437) (panel g). Human serum containing anti-Cajal antibodies was used in panel b. Human serum containing anti-EDC4 antibodies was used to detect NLS-EDC4 in (panels d and j). Rabbit anti-mCh antiserum was used to detect mCh-XRN1(1232–1706) and mCh-NLS-DDC6(289–483) (panels e and k). Rabbit anti-Myc antiserum was used to detect CCHCR1-Myc in panel h. Merge of panels a and b, d and e, g and h, j and k is shown in c, f, i, and l, respectively. DAPI staining in f and i indicate the location of nuclei. (TIF) [file pone.0282496.s004.tif]

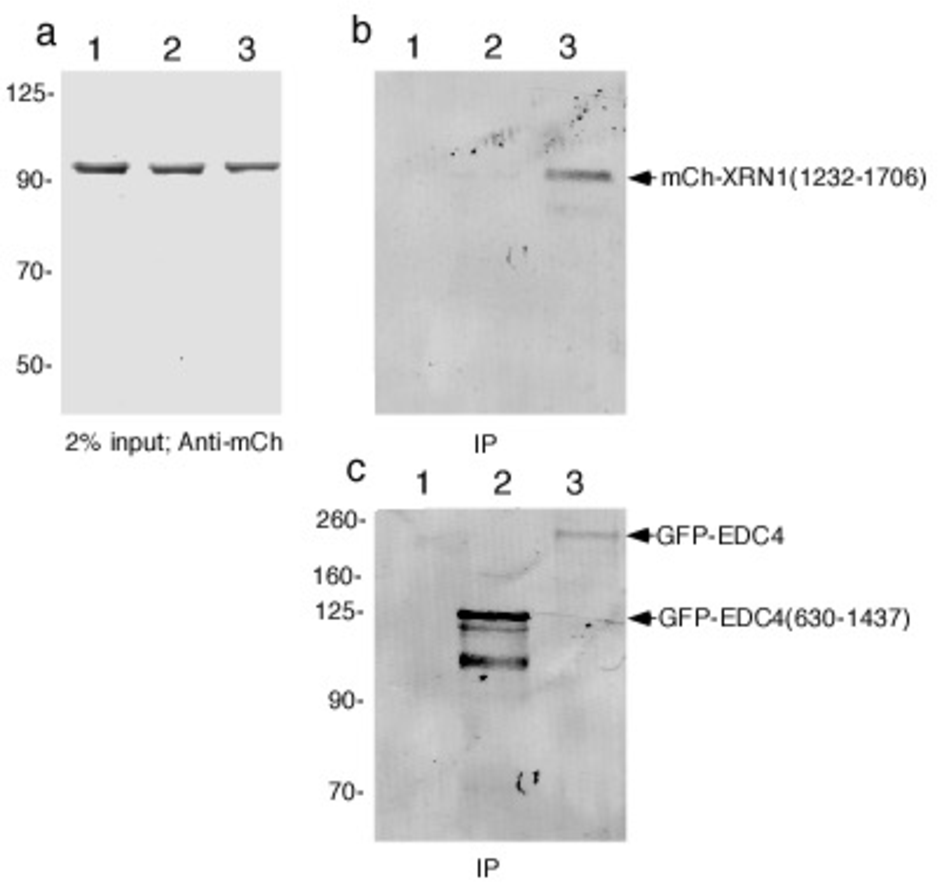

Supplement: S4 Fig — HEK293 cells were transfected with mCh-XRN1(1232–1706) alone (lane 1), mCh-XRN1(1232–1706) and GFP-EDC4(630–1437) (lane 2), or mCh-XRN1(1232–1706) and GFP-EDC4 (lane 3). Panel a shows the amount of mCh-XRN1(1232–1706) in 2% of the input protein extract. Panels b and c show that GFP-EDC4 (lane 3), but not the magnetic beads alone (lane 1) or GFP-EDC4(630–1437) (lane 2) immunoprecipitated mCh-XRN1(1232–1706). mCh-XRN1(1232–1706) was detected using rabbit anti-mCh antiserum. GFP fusion proteins were detected using mouse anti-GFP antibody. (TIF) [file pone.0282496.s005.tif]

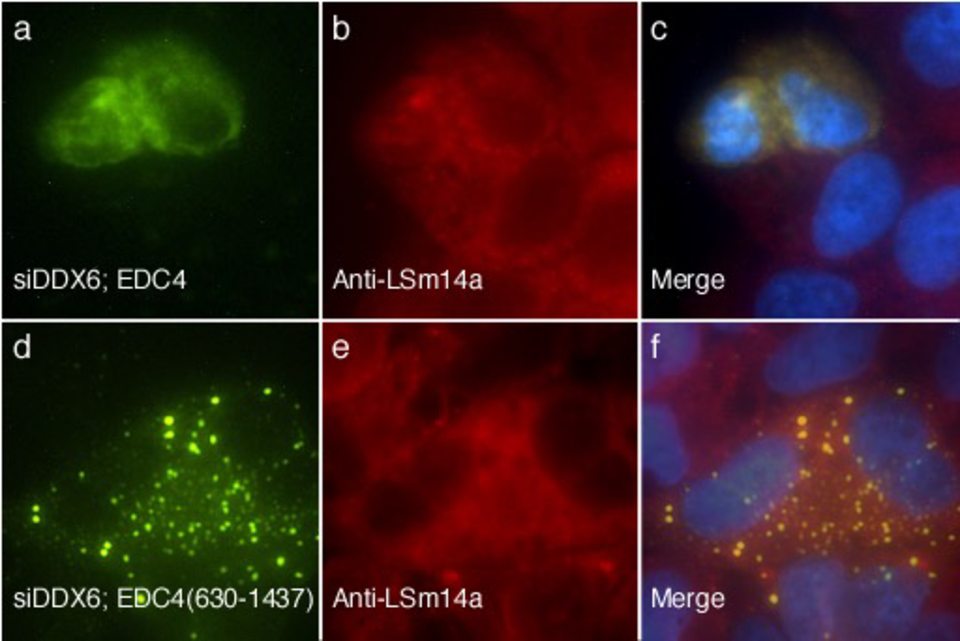

Supplement: S5 Fig — Neither full-length EDC4 nor EDC4(630–1437) was able to recruit endogenous LSm14a to cytoplasmic dots. After depletion of DDX6 using siRNA, neither GFP-EDC4 (panel a) nor endogenous LSm14a (panel b) localized to cytoplasmic dots. After depletion of DDX6, GFP-EDC4(630–1437), but not endogenous LSm14a localized to cytoplasmic dots. Mouse anti-GFP antibody was used to detect GFP-EDC4 (panel a) and GFP-EDC4(630–1437) (panel d). Rabbit anti-LSm14a antiserum was to stain for LSm14a in panels b and e. Merge of panels a and b, and d and e is shown in c and f respectively. DAPI staining in c and f indicate the location of nuclei. (TIF) [file pone.0282496.s006.tif]

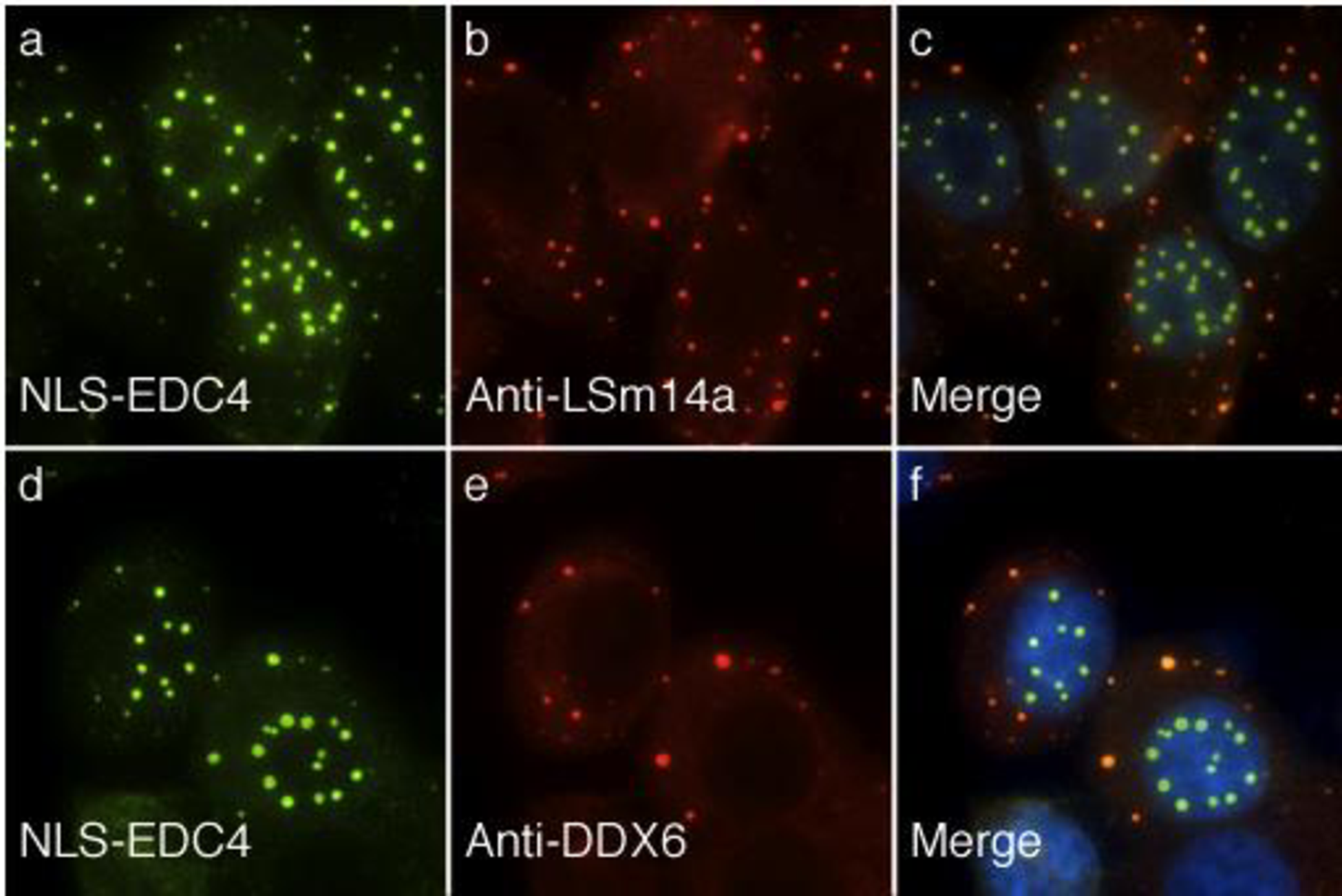

Supplement: S6 Fig — After expression of a plasmid encoding NLS-EDC4 in HEp-2 cells, human serum containing anti-EDC4 antibodies detected EDC4 in both nuclear and cytoplasmic dots (panels a and d). Endogenous LSm14a (panel b) and DDX6 (panel e) were only detected in cytoplasmic dots. Rabbit anti-LSm14a and anti-DDX6 antisera were used to detect the corresponding proteins. Merge of panels a and b, and c and e, is shown in c and f, respectively. DAPI staining in c and f indicate the location of nuclei in the preceding panels. (TIF) [file pone.0282496.s007.tif]
